# van der Waals Magnets: Material Family, Detection and Modulation of Magnetism, and Perspective in Spintronics

**DOI:** 10.1002/advs.202002488

**Published:** 2020-12-06

**Authors:** Shengxue Yang, Tianle Zhang, Chengbao Jiang

**Affiliations:** ^1^ School of Materials Science and Engineering Beihang University Beijing 100191 P. R. China

**Keywords:** detection methods, material families, modulation methods, spintronics, van der Waals magnets

## Abstract

van der Waals (vdW) materials exhibit great potential in spintronics, arising from their excellent spin transportation, large spin–orbit coupling, and high‐quality interfaces. The recent discovery of intrinsic vdW antiferromagnets and ferromagnets has laid the foundation for the construction of all‐vdW spintronic devices, and enables the study of low‐dimensional magnetism, which is of both technical and scientific significance. In this review, several representative families of vdW magnets are introduced, followed by a comprehensive summary of the methods utilized in reading out the magnetic states of vdW magnets. Thereafter, it is shown that various electrical, mechanical, and chemical approaches are employed to modulate the magnetism of vdW magnets. Finally, the perspective of vdW magnets in spintronics is discussed and an outlook of future development direction in this field is also proposed.

## Graphical abstract

van der Waals (vdW) magnets are very important in the fields of low‐dimensional physics and spintronics. A thorough summary of the latest advances in this area is provided, including the material families, various methods used in the detection and modulation of their magnetism, their potential applications in spintronics, and the opportunities and challenges in the future.

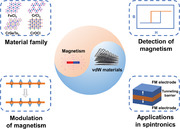

## 1 Introduction

The in‐depth control of electronic charge led to the second and third industrial revolution, while the fourth industrial revolution will be triggered by the development of spintronics based on another electronic degree of freedom, namely electron spin. The macroscopic manifestation of electron spin is magnetism, including ferromagnetism and antiferromagnetism, therefore, it is necessary to study the low‐dimensional magnetism for the effective control of spin, that is, reducing the dimensionality of magnets from 3D to 2D.

Previous works on spintronics are mainly carried out on ultrathin magnetic films prepared by epitaxial technologies, e.g., magnetron sputtering and pulsed laser deposition.^[^ ^1^ ^]^ The core of these technologies is to reduce the thickness of 3D magnets to atomic scale, but strictly speaking, these magnetic films are pseudo 2D magnets without real 2D nature because of the dangling bonds at the surface and the inevitable influences from the substrate, including lattice strain, chemical composition modification, and electronic redistribution, etc.^[^ ^2^ ^]^ In parallel, theoretical calculations have verified the presence of intrinsic magnetism in 2D material systems with sufficient anisotropy,^[^ ^3^ ^]^ and it is of great significance to develop 2D magnetic systems for understanding the fundamental issues in low‐dimensional magnetics.

The advent of van der Waals (vdW) materials provides a resolution for seeking isolated 2D magnets. Since the first successful exfoliation of monolayer graphene in 2004,^[^ ^4^ ^]^ numerous studies have been done to enrich the vdW material family. Connected by interlayer vdW interaction, surfaces and interfaces without dangling bonds can be attained in vdW materials, thus eliminating the impact of substrate. Prior researches have unveiled many fascinating electrical and optical properties of this class of materials, such as extremely high mobility^[^ ^5^ ^]^ and obvious linear dichroism.^[^ ^6^ ^]^ Besides, intensive efforts have been made to extrinsically introduce magnetism to nonmagnetic vdW materials through chemical doping,^[^ ^7^ ^]^ applying strain,^[^ ^8^ ^]^ proximity effect,^[^ ^9^ ^]^ introducing defects^[^ ^10^ ^]^ (such as vacancies, grain boundaries), and band‐structure engineering.^[^ ^11^ ^]^ The intrinsic magnetic vdW materials remain a conspicuously missing member of this family.

Until recently, the discovery of magnetic vdW materials has changed the current research status and attracted a great deal of attention. Monolayer magnetic vdW materials have been successfully prepared using micromechanical exfoliation with Scotch tape, liquid exfoliation, and molecular‐beam epitaxy (MBE).^[^ ^12^, ^13^, ^14^ ^]^ The magnetic states of intrinsic vdW magnets can be characterized by Raman spectrum, photoluminescence (PL) spectrum, second harmonic generation (SHG), spin‐polarized scanning tunneling microscopy (SPSTM), scanning single‐spin magnetometry (SSSM), magneto‐optic Kerr effect (MOKE), and magnetic circular dichroism (MCD).^[^ ^15^, ^16^, ^17^, ^18^, ^19^, ^20^ ^]^ Transport measurements based on the Hall effect (including anomalous Hall effect (AHE) and normal Hall effect (NHE)) and the tunneling magnetoresistance (TMR) effect can also be used in reading out the magnetic order in some magnetic vdW materials.^[^ ^21^, ^22^ ^]^ Due to the atomic‐scale thickness, vdW magnets are susceptible to the external perturbations. Therefore, their magnetic properties can be effectively modulated by electric field, pressure, chemical modification, strain field, etc.^[^ ^23^, ^24^, ^25^, ^26^ ^]^ Moreover, similar to other vdW materials, these vdW magnets exhibit prominent stacking‐dependent properties.^[^ ^27^ ^]^ All these phenomena show the potentials of vdW magnets in both theoretical and device applications, such as novel magneto‐electronics, magnetostriction down to the 2D limit, and the fresh superconducting phases produced by moiré patterns.

In this review, we give an exhaustive summary of the state‐of‐the‐art experimental and theoretical results on intrinsic vdW magnets. First, we list the existing magnetic vdW systems from transition metal halides to transition metal dichalcogenides (TMDs), and briefly introduce the current research status on these systems. We then present various methods of characterizing the magnetic order in magnetic vdW materials, mainly divided into optical and electrical techniques. Additionally, we summarize the possible methods to efficiently modulate the magnetism of vdW magnets, especially the newly developed mechanical routes through imposing hydrostatic pressure and external strain. Finally, we look forward to the future potentials of vdW magnets in spintronics, which will stimulate thorough investigations into this system.

## 2 The Existing and Promising Magnetic vdW Material Systems

Magnetic vdW materials are primarily categorized into transition metal halides, transition metal phosphorous tri‐chalcogenides and corresponding isostructural compounds―Cr (Si/Ge) Te_3_, ternary iron‐based tellurides, transition metal oxyhalides, and TMDs. The structural, electrical, and magnetic properties of these materials have been characterized through different methods. Here we summarize these properties of some magnetic vdW materials in **Table** **1**.

**Table 1:** Structural, electrical, and magnetic properties of some vdW magnets

Material	Space group@LT^a)^	Magnetic ordering^b)^	Electrical properties	Magnetic transition temperature^c)^	Easy axis^d)^	Spin model
Transition metal halides						
CrI_3_^[^ ^20^, ^152^ ^]^	$R\bar{3}$ (bulk) *C2/m* (f‐L)	FM (bulk/1L) a‐AFM (f‐L)	Insulator	61 K (bulk) 45 K (1L)	⊥ (Bulk/1L)	Ising
CrBr_3_^[^ ^29^, ^32^ ^]^	$R\bar{3}$	FM (bulk/1L)	Insulator	47 K (bulk) 27 K (1L)	⊥ (Bulk/1L)	Between Ising and Heisenberg
CrCl_3_^[^ ^29^, ^38^ ^]^	$R\bar{3}$ (bulk) *C2/m* (f‐L)	a‐AFM (bulk/2L)	Insulator	27 K (bulk) 16 K (2L)	∥ (Bulk/2L)	XY
VI_3_^[^ ^45^, ^46^ ^]^	$R\bar{3}$	FM	Insulator	50 K	⊥	–
FeCl_2_^[^ ^28^ ^]^	$R\bar{3}m$	a‐AFM	Insulator	24 K	⊥	Ising
FeBr_2_^[^ ^28^ ^]^	$P\bar{3}m1$	a‐AFM	Insulator	14 K	⊥	Ising
FeI_2_^[^ ^28^ ^]^	$P\bar{3}m1$	AFM	Insulator	9 K	⊥	Ising
Transition metal phosphorous tri‐chalcogenides						
FePS_3_^[^ ^54^, ^55^ ^]^	*C2/m*	AFM (bulk/1L) (zigzag‐type)	Insulator	118 K (bulk) 104 K (1L)	⊥ (Bulk/1L)	Ising
MnPS_3_^[^ ^58^ ^]^	*C2/m*	AFM (bulk/5L) (Neel‐type)	Insulator	78 K (bulk/5L)	c‐⊥ (Bulk/5L)	Heisenberg
NiPS_3_^[^ ^56^ ^]^	*C2/m*	AFM (bulk/2L) (zigzag‐type)	Insulator	155 K (bulk) Slightly lower than 155 K (2L)	∥ (Bulk/2L)	XY
FePSe_3_^[^ ^61^ ^]^	$R\bar{3}$	AFM (zigzag‐type)	Insulator	119 K	⊥	Ising
MnPSe_3_^[^ ^61^ ^]^	$R\bar{3}$	AFM (Neel‐type)	Insulator	74 K	∥	XY
CrGeTe_3_^[^ ^12^ ^]^	$R\bar{3}$	FM (bulk/2L)	Insulator	68 K (bulk) 30 K (2L)	⊥ (Bulk/2L)	Heisenberg
CrSiTe_3_^[^ ^68^ ^]^	$R\bar{3}$	FM	Insulator	32 K	⊥	Ising
Ternary iron‐based tellurides						
Fe_3_GeTe_2_^[^ ^76^ ^]^	*P6_3_/mmc*	FM (bulk/1L)	Metal	230 K (bulk) 130 K (1L)	⊥ (Bulk/1L)	Ising
Fe_4_GeTe_2_^[^ ^81^ ^]^	$R\bar{3}m$	FM (bulk/7L)	Metal	270 K (bulk/7L)	∥ (> *T*_sr_) ⊥ (< *T*_sr_) (Bulk/7L)	–
Fe_5_GeTe_2_^[^ ^83^, ^84^ ^]^	$R\bar{3}$ (Q) *R3m* (N‐C)	FM (bulk/28 nm)	Metal	275 K (28 nm) 310 K (bulk)	⊥ (Bulk/28 nm)	–
Transition metal oxyhalides						
CrOCl^[^ ^89^ ^]^	*Pmmn*	AFM	Insulator	13.5 K	⊥	–
TiOCl^[^ ^87^ ^]^	*Pmmn*	Spin‐Peierls	Insulator	91 K (PM‐IC) 48 K (IC‐sP)	–	–
TiOBr^[^ ^88^ ^]^	*Pmmn*	Spin‐Peierls	Insulator	67 K (PM‐IC) 28 K (IC‐sP)	–	–
VOCl^[^ ^90^ ^]^	*Pmmn*	AFM	Insulator	80.5 K	∥	–
FeOCl^[^ ^91^, ^96^ ^]^	*Pmmn*	AFM (bulk/f‐L)	Insulator	92 K (bulk) 14 K (f‐L)	⊥(Bulk) c‐⊥(Few‐layer)	–
Transition metal dichalcogenides						
VSe_2_^[^ ^14^ ^]^	$P\bar{3}m1$	FM (1L)	Metal	300 K (1L)	∥(1L)	–
VTe_2_^[^ ^99^ ^]^	$P\bar{3}m1$	FM (f‐L)	Metal	300 K (f‐L)	–	–
MnSe_2_^[^ ^100^ ^]^	$P\bar{3}m1$	FM (1L)	Metal	300 K (1L)	⊥ (1L)	–

^a)^ LT: low temperature, Q: quenching, N‐C: natural cooling

^b)^ FM: ferromagnetic (interlayer and intralayer), AFM: antiferromagnetic (interlayer and intralayer), a‐AFM: the magnetic structure with interlayer antiferromagnetic coupling and intralayer ferromagnetic coupling, f‐L: few‐layer, 1L: monolayer, and so on

^c)^ PM: paramagnetic phase, IC: incommensurate phase, sP: spin‐Peierls phase

^d)^ ∥: in‐plane, ⊥: out‐of‐plane, c: canted, *T*_sr_: spin‐reorientation temperature. If not specified, the properties are obtained from bulk samples.

### 2.1 Transition Metal Halides

**Figure 1:**
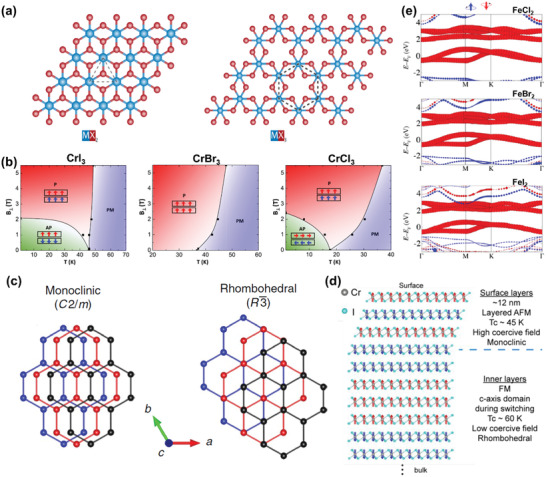
Basic properties of transition metal halides. a) The crystallographic structures of monolayer transition metal dihalides (left panel) and trihalides (right panel). Reproduced with permission.^[^ ^111^ ^]^ Copyright 2019, Springer Nature. b) The phase diagram of Cr trihalides versus temperature and magnetic field. Reproduced with permission.^[^ ^33^ ^]^ Copyright 2019, American Chemical Society. c) Two possible stacking orders of Cr trihalides. Reproduced with permission.^[^ ^24^ ^]^ Copyright 2019, Springer Nature. d) The possible spatial distribution of magnetic order and stacking order in bulk CrI_3_ from the surface layer to the inner layer. Reproduced with permission.^[^ ^35^ ^]^ Copyright 2020, American Chemical Society. e) The calculated spin‐resolved band structures of Fe dihalides. Reproduced with permission.^[^ ^42^ ^]^ Copyright 2017, American Chemical Society.

Transition metal halides are mainly composed of dihalides MX_2_ and trihalides MX_3_ (M = V, Cr, Mn, Fe, Co, Ni, Ru; X = Cl, Br, I). Arising from the relatively large radius of halide anions and the partially filled 3d electronic shells of transition metal cations, magnetic vdW materials with layered structure are expected to obtain from these compounds.^[^ ^28^ ^]^ In the dihalides, transition metal cations form triangular lattice structure in the monolayer while honeycomb lattice structure is typical for the monolayer of trihalides (**Figure** **1**a). What's more, one cation is usually surrounded by six anions to form an octahedral structure in the monolayer.

In the last few years, Cr trihalides have been widely investigated, and a series of exciting phenomena are revealed in this family of materials. As the anion changes from Cl^−^ to Br^−^ and then to I^−^, these Cr trihalides present some regular variations, including: 1) The intralayer exchange of these three compounds at the 2D limit has all proved to be ferromagnetic (FM), while the interlayer exchange changes from antiferromagnetic (AFM) to FM and again to AFM (from CrCl_3_ to CrBr_3_ and then to CrI_3_), and the corresponding magnetization direction varies from in‐plane (CrCl_3_) to out‐of‐plane (CrBr_3_ and CrI_3_).^[^ ^29^ ^]^ 2) Due to the dominant superexchange interaction^[^ ^30^ ^]^ and spin–orbit coupling (SOC),^[^ ^31^ ^]^ the Curie temperature *T*_C_ of few‐layer Cr trihalides increases from 17 K (CrCl_3_) to 37 K (CrBr_3_) then to 46 K (CrI_3_),^[^ ^29^ ^]^ deriving from the increase of both anion radius and atomic number. 3) The spin model of 2D magnetism is XY and Ising models for CrCl_3_ and CrI_3_, respectively, while it is between Heisenberg and Ising models for CrBr_3_,^[^ ^32^ ^]^ indicating the promotion of exchange anisotropy with the increase of atomic number of halide anion. In addition, the temperature‐field‐phase diagram of Cr trihalides is summarized in Figure 1b, from which it is obtained that the spin‐flip field decreases with the increase of temperature in CrI_3_ and CrCl_3_.^[^ ^33^ ^]^ The *T*_C_ of CrBr_3_ and CrCl_3_ increases as the magnetic field increases, while it is almost field‐independent in CrI_3_ due to the large anisotropy.

Moreover, the stacking order also plays an important role in the family of Cr trihalides. As shown in Figure 1c, bulk CrI_3_ will undergo a structural phase transition from a monoclinic‐stacked phase (*C2/m* space group) to a rhombohedral‐stacked phase ($R\bar{3}$ space group) when the temperature is below 210–220 K, and it exhibits ferromagnetism at low temperature.^[^ ^34^ ^]^ Conversely, 2D CrI_3_ possesses AFM interlayer coupling. Both theoretical and experimental studies shed light on the role of stacking order in the magnetism of CrI_3_, and it is found that the rhombohedral (monoclinic)‐stacked structure favors FM (AFM) interlayer coupling, resulting in the different magnetism in bulk and 2D CrI_3_.^[^ ^17^, ^27^ ^]^ To elucidate the evolution of magnetism from bulk to 2D CrI_3_, detailed investigation on bulk samples with thicknesses ranging from 25 to 200 nm has been done, which reveals a mixed magnetic structure with the AFM surface layers (≈12 nm) and the FM inner layers (Figure 1d), as proved by the two distinct transition fields in the magnetic force microscope (MFM) measurements.^[^ ^35^ ^]^ However, the presence of only one transition field confirms the pure AFM state in CrI_3_ when the thickness is reduced to below 25 nm. The emergence of mixed magnetism in bulk CrI_3_ is attributed to the different stacking order of surface and inner layers which may result from the surface effects, e.g., the surface defects generated during the preparation process or the capped interface with a *h*‐BN protective layer, consequently indicating that the variation of stacking order in 2D CrI_3_ can also be related to the preparation process and the capped *h*‐BN layer. Then the appearance of AFM magnon modes in the circular polarized Raman measurements provides another evidence for the mixed magnetic structure in bulk CrI_3_.^[^ ^36^ ^]^ Similar to CrI_3_, there is also a structural phase transition in bulk CrCl_3_ at around 240 K, below which the phase changes from monoclinic to rhombohedral.^[^ ^37^ ^]^ Nonetheless, bulk CrCl_3_ exhibits antiferromagnetism rather than ferromagnetism at low temperatures, which is different from CrI_3_. Although both 2D and bulk CrCl_3_ show identical AFM interlayer coupling, they still possess different stacking order below transition temperature (rhombohedral for bulk, monoclinic for 2D), giving rise to the huge enhancement of interlayer exchange interaction in 2D CrCl_3_ deduced from the much larger spin‐flip fields of few‐layer CrCl_3_ compared with bulk CrCl_3_.^[^ ^38^ ^]^ Besides, the stacking order‐dependent magnetism is observed in CrBr_3_ by using SPSTM as well.^[^ ^18^ ^]^ All of these introduce another way, namely the stacking order, to modulate the magnetism in this material family.

Apart from Cr trihalides, other transition metal dihalides and trihalides have also attracted surging attention recently. The intriguing properties of their bulk counterparts, such as metamagnetism^[^ ^39^ ^]^ and multiferroicity,^[^ ^40^, ^41^ ^]^ make them possible to have marvelous characteristics at 2D limit. As predicted by theoretical calculations, Fe dihalides―FeCl_2_, FeBr_2_, and FeI_2_ will become spin half metals with large spin gaps when the thickness is reduced to monolayer, which is revealed by the presence of only spin‐minority bands at around Fermi level in the calculated spin‐polarized electronic band structures (Figure 1e).^[^ ^42^ ^]^ Hereafter, the ground states in these compounds are related to the extra orbital splitting caused by the trigonal distortions around Fe‐site.^[^ ^43^ ^]^ Then, the theoretical analyses disclose the excellent spin transport properties in Fe dihalides.^[^ ^44^ ^]^ Besides, a typical trihalide―VI_3_ has also been widely studied, which is a FM semiconductor. Both bulk and few‐layer VI_3_ are successfully obtained by different groups.^[^ ^45^, ^46^ ^]^ According to the analysis of phase transition behavior, a structural transition (*T*_s_ ≈ 79 K) and two magnetic transitions (*T*_C_ ≈ 50 K, *T*_m_ ≈ 30 K) coexist in bulk VI_3_,^[^ ^47^ ^]^ where the latter magnetic transition at 30 K is accompanied by another structural transition,^[^ ^48^ ^]^ indicating the presence of magnetoelastic coupling. Also, the theoretical studies demonstrate that the magnetic properties of 2D VI_3_ strongly depend on the stacking order,^[^ ^49^ ^]^ which is similar to CrI_3_. Due to the strong magnetoelastic coupling and the stacking‐dependent magnetism, it is possible to modulate the magnetism of 2D VI_3_ via applying pressure or suitable strain.

In addition, various phenomena such as large valley splitting,^[^ ^50^ ^]^ higher‐spin Kitaev model,^[^ ^51^ ^]^ and quantum anomalous Hall effect,^[^ ^52^ ^]^ have been reported in transition metal halides, suggesting their potentials in different research fields.

### 2.2 Transition Metal Phosphorous Tri‐Chalcogenides

**Figure 2:**
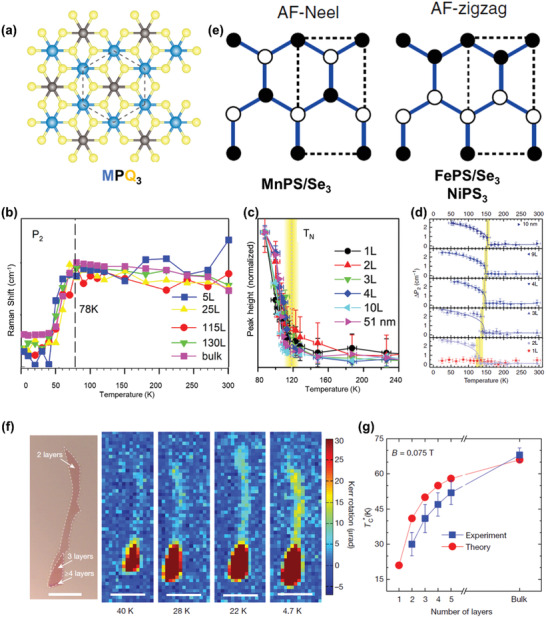
Basic properties of transition‐metal phosphorous tri‐chalcogenides and the isostructural Cr(Si/Ge)Te_3_. a) The crystal structure of these isostructural compounds. Reproduced with permission.^[^ ^111^ ^]^ Copyright 2019, Springer Nature. b) The temperature dependence of peak positions for *P*_2_ mode in MnPS_3_ with different thicknesses. Reproduced with permission.^[^ ^58^ ^]^ Copyright 2019, American Chemical Society. c) The temperature dependence of peak intensities for the magnetic‐order induced *P*_1a_ mode in FePS_3_ with different thicknesses. Reproduced with permission.^[^ ^55^ ^]^ Copyright 2016, American Chemical Society. d) The temperature dependence of peak splitting for *P*_2_ mode in NiPS_3_ with different thicknesses. Reproduced with permission.^[^ ^56^ ^]^ Copyright 2019, Springer Nature. e) The schematic diagram of Neel‐type and zigzag‐type magnetic structures for MPQ_3._ Reproduced with permission.^[^ ^60^ ^]^ Copyright 2015, American Physical Society. f) Optical micrograph of few‐layer CrGeTe_3_ and the respective characterization of magnetism using MOKE at different temperatures. Scale bar: 10 µm. g) The *T*_C_ of CrGeTe_3_ versus number of layers. Reproduced with permission.^[^ ^12^ ^]^ Copyright 2017, Springer Nature.

Researches on transition metal phosphorous tri‐chalcogenides can date back to late 1800s,^[^ ^53^ ^]^ and the enthusiasm has been reignited due to their lamellar structures and plentiful functionalities. To describe the crystal structure more clearly, the abbreviation of this material family is best written as [M_1_M_2_] [P_2_(Q)_6_] for the common sublattice [P_2_(Q)_6_] in the basal plane, and in this formula Q represents S or Se atoms, while M_1_ and M_2_ are transition metal atoms. The monolayer shows a sandwich structure, where the top and bottom S (Se) atoms constitute octahedral gaps, 2/3 of which are filled with M_1_/M_2_ atoms and the rest are filled with P–P dimers. M_1_/M_2_ atoms are arranged in a honeycomb lattice (**Figure** **2**a), similar to MX_3_. Based on the atomic species and chemical valences of M_1_ and M_2_, the crystal structure can be further subdivided,^[^ ^53^ ^]^ which is not the emphasis of this review. Herein, we focus on the materials with the same atomic species and chemical valences for M_1_ and M_2_, and then the abbreviation of MPQ_3_ (M = Fe, Ni, Mn) will be used in the latter parts.

Corresponding to Cr trihalides, the magnetic properties of MPQ_3_ display strong dependence on the choice of M atoms. As for the sulfides, with M changes from Mn to Fe and then to Ni, the spin model of 2D magnetism varies from Heisenberg to Ising and then to XY types,^[^ ^54^, ^55^, ^56^ ^]^ while the Neel temperature *T*_N_ of bulk MPQ_3_ is from ≈78 to 123 and then to 155 K,^[^ ^57^ ^]^ respectively. As shown in Figure 2b–d, the transition temperature of MnPS_3_, FePS_3_, and NiPS_3_ with different thicknesses is characterized by the Raman measurements, presenting little dependence on thickness.^[^ ^55^, ^56^, ^58^ ^]^ What needs to be emphasized is that all the MPQ_3_ materials exhibit antiferromagnetism at low temperature. In contrast to ferromagnets, the magnetic structures of antiferromagnets are far more complicated. For example, MnPS_3_ possesses a Neel‐type magnetic structure, while both FePS_3_ and NiPS_3_ have zigzag‐type magnetic structures (Figure 2e).^[^ ^59^, ^60^ ^]^ In particular, the Neel‐type magnetic structure in MnPS_3_ breaks both time and spatial reversal symmetry at the AFM state, resulting in linear magnetoelectric responses, which is verified using SHG in the recent research.^[^ ^57^ ^]^ The same phenomenon is also expected from its selenide counterpart, MnPSe_3_, which shares an identical magnetic structure with MnPS_3_.^[^ ^61^ ^]^ However, the magnetization direction shifts from out‐of‐plane for MnPS_3_ to in‐plane for MnPSe_3_, subsequently causing the transformation of spin model from Heisenberg to XY types. The *T*_N_ of bulk MnPSe_3_ is then estimated to be 74 K using the neutron diffraction. As for FePSe_3_, it is an Ising‐type antiferromagnet with a zigzag magnetic structure, and the *T*_N_ of bulk FePSe_3_ is around 119 K, close to FePS_3_. Compared with the sulfides, studies on 2D selenides are inadequate, and there is plenty of room to investigate the selenides to the 2D limit.

Because of zero net‐magnetization, it is particularly difficult to detect the magnetic order in antiferromagnets. For 2D MPQ_3_, plenty of works have focused on the indirect probe of magnetic order using Raman spectrum (Figure 2b–d), and the *T*_N_ at 2D limit is obtained through analyzing the phonon anomaly or the peak splitting in the temperature‐dependent Raman spectra.^[^ ^55^, ^58^, ^62^, ^63^ ^]^ Besides, the existence of magnon mode in 2D FePS_3_^[^ ^63^ ^]^ and the long‐distance magnon transport^[^ ^64^ ^]^ in 2D MnPS_3_ reveal their potentials in magnonics. Moreover, the theoretically predicted electrical control of valley polarization^[^ ^65^ ^]^ in monolayer MnPSe_3_ and the coupling of AFM order to valley order^[^ ^66^ ^]^ in monolayer MnPS_3_ make them possible for the applications in valleytronics.

In stark contrast, chromium‐based tellurides Cr(Si/Ge)Te_3_, isostructural compounds of MPQ_3_, are typical ferromagnets at finite temperature. Among them, bilayer CrGeTe_3_ is the first experimentally observed vdW magnet with intrinsic long‐range FM order at 2D limit, which is characterized by MOKE measurements (Figure 2f).^[^ ^12^ ^]^ Though the maintenance of magnetic order cannot be realized in the monolayer, it is still a landmark of vdW magnets, and the suppression of magnetic order in monolayer CrGeTe_3_ has been interpreted by the transcendence of magnetic dipolar anisotropy energy over magneto‐crystalline anisotropy energy.^[^ ^67^ ^]^ CrGeTe_3_ is a typical Heisenberg‐type ferromagnet, and its *T*_C_ decreases from about 68 K for bulk samples to 30 K for bilayer samples (Figure 2g). However, quasi‐2D Ising‐type magnetization behavior has been observed in bulk CrSiTe_3_ with a *T*_C_ of ≈33 K,^[^ ^68^ ^]^ while the magnetization behavior at 2D limit is rarely reported. To explore the relationship between electronic structure and magnetism, measurements such as angle‐resolved photoemission spectroscopy are carried out on CrGeTe_3_, which reveal the importance of interaction between 5p orbitals in Te atom and *e*_g_ orbitals in Cr atom to the formation of FM order.^[^ ^69^ ^]^ In parallel, homologous studies on CrSiTe_3_ unveil the intimate relationship between the superexchange coupling and the electronic structure.^[^ ^70^ ^]^ At the same time, the strong electronic correlations in CrSiTe_3_ weaken interlayer exchange, resulting in uncertain FM or AFM interlayer exchange coupling, which needs further experimental verifications. Moreover, the presence of strong spin‐phonon coupling has been confirmed both experimentally and theoretically in CrGeTe_3_ and CrSiTe_3_,^[^ ^71^, ^72^, ^73^, ^74^, ^75^ ^]^ suggesting the possible modulation of magnetism through pressure or strain.

### 2.3 Ternary Iron‐Based Tellurides

**Figure 3:**
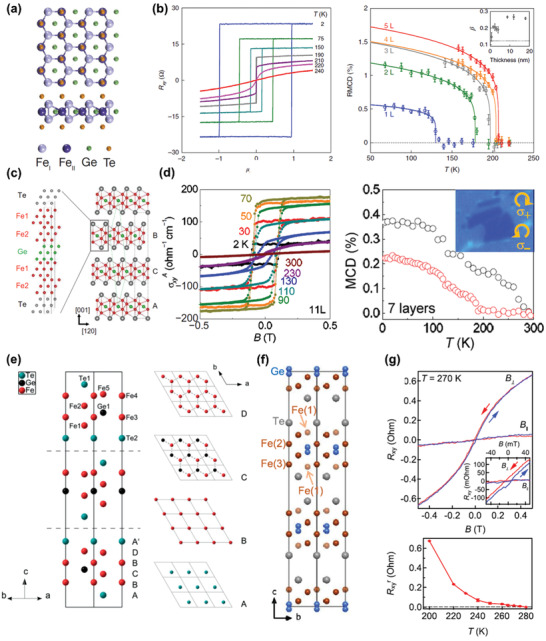
Basic properties of ternary iron‐based tellurides. a) The crystal structure of Fe_3_GeTe_2_. Reproduced with permission.^[^ ^21^ ^]^ Copyright 2018, Springer Nature. b) The magnetism characterization of Fe_3_GeTe_2_. The left panel: the field‐dependent *R_xy_* measured on a 12 nm thick sample at different temperatures. The right panel: temperature‐dependent MCD measurements of Fe_3_GeTe_2_ with different thicknesses (dots) and the corresponding fitting results using critical power law (lines). The inset shows the derived critical exponent *β* as a function of thickness. Reproduced with permission.^[^ ^76^ ^]^ Copyright 2018, Springer Nature. c) The crystal structure of Fe_4_GeTe_2_. d) The magnetism characterization of Fe_4_GeTe_2_. The left panel: the anomalous Hall conductivity versus sweeping magnetic fields for an 11‐layer sample under different temperatures. The right panel: temperature‐dependent MCD measurements on a 7‐layer sample with (black circles)/without (red circles) a 0.5 T out‐of‐plane field, while the inset displays the optical micrograph of the seven‐layer sample and the circular polarization configuration for MCD measurements. Reproduced with permission.^[^ ^81^ ^]^ Copyright 2020, American Association for the Advancement of Science. e,f) Two existing crystal structures of Fe_5_GeTe_2_. Reproduced with permission.^[^ ^84^ ^]^ Copyright 2018, Wiley‐VCH. g) The magnetism characterization of a 28 nm thick Fe_5_GeTe_2_ sample. Top panel: the hysteresis loop measured using AHE at 270 K with external magnetic field vertical/parallel to the basal plane of sample, and the inset is a magnification at around zero field. Bottom panel: the temperature dependence of remnant Hall resistance at a temperature range of 200–280 K. Reproduced with permission.^[^ ^83^ ^]^ Copyright 2019, American Chemical Society.

The crystal structure of ternary iron‐based tellurides Fe*_y_*GeTe_2_ (*y* = 3, 4, 5) is much more complicated compared to forenamed magnetic vdW materials. Among them, Fe_3_GeTe_2_ is the first material to be investigated at 2D limit,^[^ ^76^ ^]^ with covalently bonded Fe_3_Ge slabs sandwiched between two Te atomic layers to form a monolayer composed of five atomic sublayers (**Figure** **3**a). Unlike MX_2_, MX_3_, and MPQ_3_, which behave like insulators or semiconductors, Fe_3_GeTe_2_ shows metallic properties, suggesting that it will possess itinerant ferromagnetism. According to the analysis of rectangular hysteresis loop at low temperature and critical behavior, it is confirmed that monolayer Fe_3_GeTe_2_ is a 2D Ising system with a critical exponent of 0.12–0.16 (Figure 3b), close to 0.125 for 2D Ising model. Due to the relatively large magnetic anisotropy comparable to CoFeB magnetic films,^[^ ^21^ ^]^ the *T*_C_ of monolayer Fe_3_GeTe_2_ reaches up to 130 K,^[^ ^76^ ^]^ much higher than monolayer CrI_3_ (45 K)^[^ ^20^ ^]^ and bilayer CrGeTe_3_ (30 K).^[^ ^12^ ^]^ Moreover, the crystal structure refinements and theoretical calculations have verified that small amounts of Fe vacancies exist in Fe_3_GeTe_2_, making this compound an intermetallic with a small phase width.^[^ ^77^, ^78^ ^]^ To describe the variation of Fe content in this compound, it is best written as Fe_3−_ *_x_*GeTe_2_, and we here use Fe_3_GeTe_2_ as a simplification. The magnetic properties of Fe_3_GeTe_2_ are strongly dependent on Fe content (details in Section 4.3).^[^ ^79^ ^]^ Combined with the itinerant magnetism, methods such as alloying^[^ ^80^ ^]^ and ionic gating^[^ ^21^ ^]^ are therefore very useful for the modulation of its magnetism.

Following the idea of composition engineering with changing Fe content, researchers have theoretically investigated the suitable Fe content and found that Fe_4_GeTe_2_ and Fe_5_GeTe_2_ are energetically favorable with vdW‐connected atomic layers,^[^ ^81^ ^]^ enabling further studies on these compounds at 2D limit. Fe_4_GeTe_2_ has been successfully synthesized very recently, which has an even more complicated crystal structure than Fe_3_GeTe_2_ (Figure 3c). It is composed of Fe_4_Ge sublattices sandwiched between two Te atomic layers, where the sublattices involve one Ge atomic sublayer and four Fe atomic sublayers with Ge atoms locating in the middle of a corrugated hexahedral made of Fe–Fe dumbbells. As a result, each single layer contains seven atomic sublayers as proved by microscopic analysis. Because of its complex structure, the obtained thinnest sample can only be seven layers up to now, with a *T*_C_ of about 270 K. Based on the theoretical calculations, the exchange interaction is enhanced with the increase of Fe content, accounting for the relatively high *T*_C_ in Fe_4_GeTe_2_. Additionally, Fe_4_GeTe_2_ also exhibits itinerant ferromagnetism, revealed by the pronounced hysteresis loop at low temperature (Figure 3d). Moreover, a spin‐reorientation temperature (*T*_sr_) of ≈210 K is discovered in this material, below which the magnetic anisotropy turns from in‐plane to out‐of‐plane, resulting from the combined effect of magneto‐crystalline anisotropy (*K*_m_) and shape anisotropy (*K*_s_). *K*_m_ favors out‐of‐plane anisotropy and dominates at low temperature, while *K*_s_ supports in‐plane anisotropy and it is predominant at high temperature, leading to the transition of magnetic anisotropy with temperature. As shown in the right panel of Figure 3d, at the temperature above *T*_sr_, MCD signal is nearly zero without field, but it presents a considerable value under out‐of‐plane field, indicating that the moments are intrinsically aligned in‐plane because MCD can only detect out‐of‐plane magnetization.^[^ ^82^ ^]^ Then, the magnetization direction switches to out‐of‐plane with the decrease of temperature, which is verified by the large MCD intensity without field.^[^ ^81^ ^]^ Hereafter, further studies are needed to clarify the magnetism of monolayer Fe_4_GeTe_2_. As for Fe_5_GeTe_2_, the crystal structure has not been unambiguously characterized due to intrinsic disorder and short‐range order.^[^ ^83^ ^]^ What's more, the preparation process displays huge impacts on the crystal structure,^[^ ^83^, ^84^ ^]^ and two possible crystal structures under different synthesis methods are listed in Figure 3e,f, of which the space groups are *R3m* and $R\bar{3}m$, respectively. The existence or not of split sites of Fe (1) atoms and Ge atoms is the major difference between these two structures.^[^ ^85^ ^]^ Likewise, the exfoliated thinnest Fe_5_GeTe_2_ flakes are around 10 nm thick, and the *T*_C_ ranges from 270 to 300 K for samples with different thicknesses.^[^ ^83^ ^]^ The bottom panel of Figure 3g shows one of the temperature‐dependent AHE measurement results, and it is known that the *T*_C_ is around 275 K for a 28 nm thick sample by the appearance of non‐zero remnant anomalous Hall resistance. Besides, the anomalous Hall resistance of Fe_5_GeTe_2_ exhibits a hysteric behavior in the field‐dependent AHE measurements, and the hysteresis loop is more distinct under out‐of‐plane fields than in‐plane fields, implying the prominent itinerant ferromagnetism with an out‐of‐plane easy axis in Fe_5_GeTe_2_ (top panel of Figure 3g). In addition, the magnetic transition behaviors of this compound are particularly complex, in which possible intermediate states including spin‐canting, spin‐reorientation, and helical‐spin state^[^ ^83^, ^85^ ^]^ are likely to exist, and strong magnetoelastic coupling is also observed.^[^ ^85^ ^]^ Furthermore, studies concerning the dependence of magnetism on Fe content, alloying, and synthesis process are expected for completing the phase diagram of ternary iron‐based tellurides.

### 2.4 Transition Metal Oxyhalides

**Figure 4:**
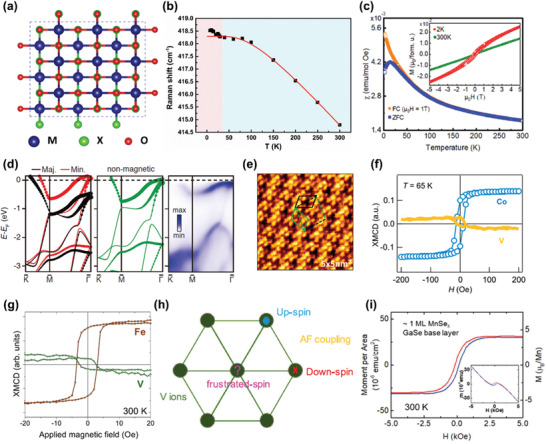
Basic properties of transition metal oxyhalides and transition metal dichalcogenides. a) The crystal structure of transition metal oxyhalides. b) Temperature dependence of the peak position for *A*_g_^2^ mode in CrOCl (black dots), and the anharmonic fitting results (red line). Reproduced with permission.^[^ ^93^ ^]^ Copyright 2019, American Chemical Society. c) The temperature‐dependent magnetic susceptibility measured on chemically exfoliated FeOCl nanoflakes using the field‐cooling mode (blue, magnetic field: 1T) and zero‐field‐cooling mode (orange), and the inset shows the field‐dependent magnetization measured at 2 and 300 K. Reproduced with permission.^[^ ^96^ ^]^ Copyright 2020, American Chemical Society. d) The calculated spin‐splitting (left, where red and black denotes the majority and minority spins, respectively), nonmagnetic (middle), and the experimentally observed (right) electronic structures of monolayer VSe_2_. Reproduced with permission.^[^ ^101^ ^]^ Copyright 2018, American Chemical Society. e) The atomic‐resolution scanning tunneling microscopy image of monolayer VSe_2_ grown on MoS_2_ substrate. Reproduced with permission.^[^ ^102^ ^]^ Copyright 2019, American Chemical Society. f) The elemental‐resolved hysteresis loops of Co (blue) and V (yellow) measured on the MBE‐grown monolayer VSe_2_ capped by a Co layer using XMCD at 65 K. Reproduced with permission.^[^ ^105^ ^]^ Copyright 2019, American Chemical Society. g) The elemental‐resolved XMCD signal of Fe (red) and V (green) as a function of magnetic field measured on the MBE‐grown monolayer VSe_2_ capped by a Fe layer measured at 300 K. Reproduced with permission.^[^ ^106^ ^]^ Copyright 2019, American Physical Society. h) Schematic diagram of spin frustration in VSe_2_. Reproduced with permission.^[^ ^107^ ^]^ Copyright 2019, Wiley‐VCH. i) The measured hysteresis loop of monolayer MnSe_2_ grown on GaSe substrate at 300 K, and the inset shows the raw data without the subtraction of diamagnetic background. Reproduced with permission.^[^ ^100^ ^]^ Copyright 2018, American Chemical Society.

Bulk materials of transition metal oxyhalides MOX (M = Ti, V, Cr, Fe; X = Cl, Br) have been widely studied since 1970s,^[^ ^86^ ^]^ which are known to be quasi‐2D material systems with atomic slabs stacking along *c* axis connected by vdW force. The basal plane is made up of corrugated MO double atomic layers sandwiched between two halogen atom layers. In aforementioned systems, transition metal atoms are arranged in triangular or honeycomb lattices, while rectangular arrangements occur in this material family (**Figure** **4**a). With different number of 3d electrons in transition metal cations, these isostructural compounds show plentiful phase transition behaviors and magnetic structures in the bulk form. TiOCl experiences two phase transitions at 91 and 48 K, from a normal paramagnetic phase to an incommensurate phase and then to a spin Peierls phase.^[^ ^87^ ^]^ Similar phase transition behaviors exist in TiOBr with transition temperatures at around 67 and 28 K, respectively.^[^ ^88^ ^]^ Both of them show quasi‐1D magnetism at low temperature.^[^ ^87^, ^88^ ^]^ Strong magnetoelastic coupling is observed in VOCl (CrOCl) at the *T*_N_ of 80.5 K (13.5 K).^[^ ^89^, ^90^ ^]^ Below *T*_N_, VOCl has a twofold magnetic superstructure with an easy plane, while CrOCl holds a fourfold magnetic superstructure with an easy axis along *c* axis. Additionally, a phase transition with unclear origin at around 27.2 K occurs in CrOCl.^[^ ^89^ ^]^ FeOCl has an incommensurate modulated AFM ground state with a *T*_N_ of 92 K, and strong magnetoelastic coupling also presents in this compound.^[^ ^91^ ^]^

Recently, the theoretical prediction of FM ground state for monolayer CrOCl has reintroduced this material family to vdW magnets.^[^ ^92^ ^]^ Strong spin‐phonon coupling in few‐layer CrOCl is revealed by the phonon anomaly at around 27 K from the temperature‐dependent Raman spectra (Figure 4b).^[^ ^93^ ^]^ Besides, CrOCl presents optical linear dichroism and ambient stability at atomic scale. In addition, the *T*_C_ of monolayer CrOCl is largely increased by isoelectronic atomic substitution, which is indicated by the theoretical calculations.^[^ ^94^, ^95^ ^]^ For example, the theoretically predicted *T*_C_ of monolayer CrSCl and CrSeBr are both around 500 K, much higher than the predicted *T*_C_ of monolayer CrOCl (≈160 K).^[^ ^92^, ^95^ ^]^ As for FeOCl, few‐layer samples are obtained by chemical exfoliation, which display intrinsic antiferromagnetism like its bulk counterparts with a decrease of *T*_N_ from 92 K (bulk) to 14 K.^[^ ^96^ ^]^ However, according to the different temperature dependence of magnetic susceptibility in the field‐cooling and zero‐field‐cooling modes at low temperature as well as the hysteresis at 2 K, FeOCl nanosheets are likely to possess canted antiferromagnetism (Figure 4c). Additionally, VOCl nanoflakes are synthesized using chemical vapor deposition (CVD), and the fabricated memristive devices based on VOCl nanoflakes show their potentials in high performance electronics.^[^ ^97^ ^]^ In brief, abundant room remains here to explore the magnetism and phase transition behaviors of MOX at 2D limit.

### 2.5 Transition Metal Dichalcogenides

TMDs are a huge class of vdW materials, exhibiting various electrical and optical properties,^[^ ^98^ ^]^ while the recent introduction of magnetism to this family has aroused extensive researches. The experimentally synthesized magnetic TMDs, MQ_2_ (M = V, Mn; Q = Se, Te), all crystallize in the 1T structure,^[^ ^14^, ^99^, ^100^ ^]^ which are isostructural to MX_2_. Among these, monolayer VSe_2_ is the first one to display magnetism at 2D limit, yet this finding remains controversial up to now. At beginning, room‐temperature ferromagnetism with an easy plane is observed in both chemically exfoliated^[^ ^13^ ^]^ and MBE‐grown monolayer VSe_2_^[^ ^14^ ^]^ using magnetometer. However, further analysis on electronic band structure of monolayer VSe_2_ proclaims the absence of spin‐polarized bands (Figure 4d), which is in contradiction with the presence of ferromagnetism.^[^ ^101^ ^]^ On the basis of magnetism characterization through X‐ray magnetic circular dichroism (XMCD) and theoretical calculations, the possibility of FM ground state in monolayer VSe_2_ is ruled out,^[^ ^101^, ^102^, ^103^ ^]^ and this state is suppressed by the charge density wave state at low temperature (Figure 4e). Nevertheless, the energy difference between these two states is very small,^[^ ^104^ ^]^ making monolayer VSe_2_ at the verge of phase transition. This opinion is confirmed by the long‐range magnetic order in monolayer VSe_2_ induced by interfacial hybridization^[^ ^105^ ^]^ or magnetic proximity effects (MPE).^[^ ^106^ ^]^ As illustrated in Figure 4f,g, the V loop shows hysteric characteristics in the element‐specific XMCD measurements on VSe_2_/Co and VSe_2_/Fe heterostructures, which is a clear proof for the ferromagnetism in VSe_2_. Beyond that, the presence of spin frustration^[^ ^107^ ^]^ in monolayer VSe_2_ is another evidence for the unstable magnetic state, which possibly arises from the triangular spin structure where the mutual anti‐alignment of spin cannot be realized (Figure 4h). So far, the mechanism of robust magnetism in monolayer VSe_2_ is not completely understood, and the dimensionality effect^[^ ^101^ ^]^ together with atomic‐scale structural deformation^[^ ^107^ ^]^ may play a role in clarifying its nature. As for the corresponding telluride―VTe_2_, multilayer samples with thicknesses ranging from 8 to 60 nm are prepared by CVD.^[^ ^99^ ^]^ These samples are characterized by magnetometer, which show ferromagnetism. The signal measured by magnetometer is a collection of all samples on the substrate, while the transport measurements on individual multilayer specimens present no signal of long‐range magnetic order but the Kondo effect caused by the localized order,^[^ ^108^ ^]^ resulting from the spontaneously formed interstitial V ions, just as the case of V_5_Se_8_.^[^ ^109^ ^]^ Furthermore, MBE‐grown monolayer VTe_2_ does not show ferromagnetism through the magnetism characterization using XMCD,^[^ ^110^ ^]^ which is in coincidence with VSe_2_. Therefore, extensive researches are needed to interpret the experimentally observed magnetism in VTe_2_. Another one of magnetic TMDs is MnSe_2_, and the magnetism of MBE‐grown monolayer MnSe_2_ is characterized using magnetometer, presenting room‐temperature magnetism with an out‐of‐plane easy axis, as shown by the obvious hysteresis of monolayer MnSe_2_ at room temperature (Figure 4i).^[^ ^100^ ^]^ Nevertheless, the magnetism in thicker samples may come from the integration of monolayer MnSe_2_ and/or interface of non‐vdW *α*‐MnSe (111). Afterward, it is required to investigate the magnetism of MnSe_2_ using different methods and clarify its origin.

**Figure 5:**
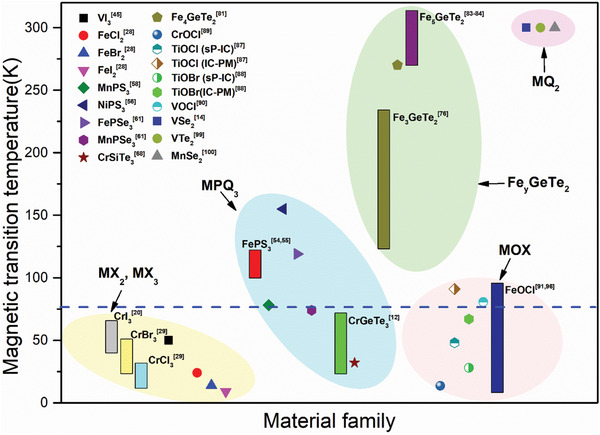
The magnetic transition temperature of the materials mentioned in this work. The bar means that the transition temperature of this material is different for different forms (e.g., the bulk and few‐layer samples). sP: Spin‐Peierls phase, IC: incommensurate phase, PM: paramagnetic phase. The blue dashed line denotes the liquid nitrogen temperature. For details, please refer to the corresponding part in this review.

At the end of this part, we present the comparison between the magnetic transition temperatures of the materials aforementioned. As shown in **Figure** **5**, the magnetic transition temperatures of most materials are well below room temperature, and many of them are even lower than the liquid nitrogen temperature. Obviously, magnetic vdW materials with high transition temperature are urgently demanded.

## 3 Detection of Magnetism in vdW Magnets

With the development and modification of existing technologies and instruments, several techniques have been employed to characterize the magnetism of vdW materials, which are mainly divided into optical and electrical methods. Herein, we will give a thorough introduction of currently used techniques in the characterization of vdW magnets and their physical mechanisms. We also provide a systematic comparison between these detection methods in **Table** **2**, including the application range and feature of different detection methods.

**Table 2:** The comparison between different detection methods

		Magnetic ordering			Magnetization direction		
	Detection tools	a‐AFM^a)^	FM	AFM	IP	OP	Remarks
Optical techniques	MOKE MCD	Y	Y	N	N	Y	Direct observation of magnetism Convenient
	XMCD	Y	Y	N	Y	Y	Element‐resolvable Can detect the inner‐shell magnetic transitions Require synchrotron radiation source
	PL	Y	Y	N	N	Y	Indirect methods Combine valleytronics with spintronics
	Raman	Y	Y	Y	Y	Y	Indirect methods Supportive tools for the direct methods
	SHG	Y	N	Y	U	Y	Sensitive to the symmetry (applicable for studying the stacking order) Require magnetic structures without inversion center
	SSSM	Y	Y	N	U	Y	Quantitative analysis Nanoscale spatial resolution Require further data processing
Electrical techniques	MTJ‐based measurements	Y	Y	Y	Y	Y	Compatible with devices Not suitable for FM insulators
	SPSTM	Y	Y	N	U	Y	Microscopic analysis simultaneously
	AHE‐based measurements	U	Y	N	N	Y	Compatible with devices
	Hall micro‐magnetometry	Y	Y	N	N	Y	Quantitative analysis

^a)^ FM: ferromagnetic (interlayer and intralayer), AFM: antiferromagnetic (interlayer and intralayer), a‐AFM: the magnetic structure with interlayer antiferromagnetic coupling and intralayer ferromagnetic coupling, IP: in‐plane direction, OP: out‐of‐plane direction, Y: yes, N: no, U: uncertain, which means that this needs more experimental verification.

### 3.1 Optical Methods

#### 3.1.1 MOKE and MCD Measurements

**Figure 6:**
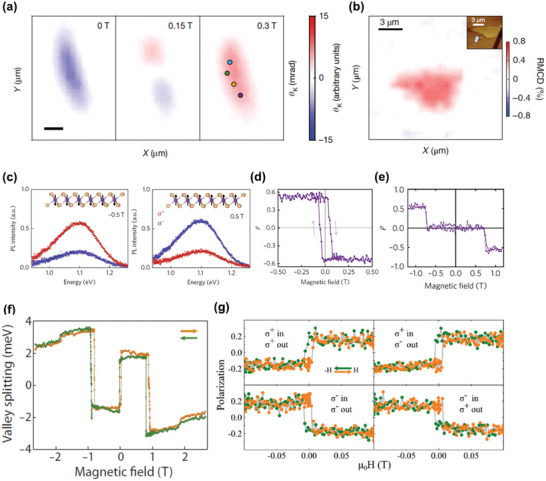
Detection of magnetism using MOKE, MCD, and PL. a) Direct observation of ferromagnetism in monolayer CrI_3_ using MOKE and the evolution of magnetic domain structures with sweeping the fields. Reproduced with permission.^[^ ^20^ ^]^ Copyright 2017, Nature Publishing Group. b) Direct observation of ferromagnetism in monolayer Fe_3_GeTe_2_ using MCD. Reproduced with permission.^[^ ^76^ ^]^ Copyright 2018, Springer Nature. c) The PL intensities of right circularly polarized light (red) and left circularly polarized light (blue) in monolayer CrI_3_ with the magnetic moments aligned downward (left panel) and upward (right panel), while the incident light is always linearly polarized. d) The field‐dependent circular polarization of collected lights in a monolayer CrI_3_. e) The circular polarization of collected lights versus sweeping the magnetic fields in a bilayer CrI_3_. Reproduced with permission.^[^ ^113^ ^]^ Copyright 2017, Springer Nature. f) The field‐dependent proximity‐induced valley splitting in a CrI_3_/WSe_2_ heterostructure. Reproduced with permission.^[^ ^114^ ^]^ Copyright 2017, American Association for the Advancement of Science. g) The field‐dependent relative polarization of collected luminescence of monolayer CrBr_3_ under various polarization configurations for excitation and collection lights, where *σ* ^+^ denotes right circularly polarized light and *σ* ^−^ is left circularly polarized light. Reproduced with permission.^[^ ^16^ ^]^ Copyright 2019, American Chemical Society.

The FM order in vdW magnets can be directly identified through MOKE or MCD measurements,^[^ ^12^, ^20^, ^21^, ^29^ ^]^ and these two methods detect the change of polarization and ellipticity of incident linearly polarized laser, respectively. The former change originates from different optical refractive index for right and left circularly polarized light induced by the magnetization of sample, while the latter change is related to different absorption for right and left circularly polarized light.^[^ ^111^ ^]^ Limited by instrument settings and signal strength, the currently used MOKE and MCD measurements can only recognize the out‐of‐plane magnetization of vdW magnets at 2D limit, thus they have been extensively used in the characterization of Ising‐type and Heisenberg‐type vdW ferromagnets, such as CrI_3_,^[^ ^23^ ^]^ CrBr_3_,^[^ ^29^ ^]^ CrGeTe_3_,^[^ ^112^ ^]^ and Fe_3_GeTe_2_.^[^ ^21^ ^]^ The magnetism of CrI_3_ has been characterized using MOKE. As shown in **Figure** **6**a, monolayer CrI_3_ is spontaneously magnetized at first (spin down, denoted by color blue). Then, spins in the upper part of the flake flip at an external magnetic field of 0.15 T, while the whole sample is spin up at 0.3 T (denoted by color red), suggesting the formation of magnetic domains with different coercivity.^[^ ^20^ ^]^ Besides, the ferromagnetism of monolayer Fe_3_GeTe_2_ is verified by the considerable MCD intensity^[^ ^76^ ^]^ (Figure 6b). Involving the real part rather than the imaginary part of optical conductivity, MCD is more advantageous than MOKE, because the measurement results of MCD are less affected by the birefringence and interference in both optical lens system and different interfaces in comparison with MOKE.^[^ ^111^ ^]^

Replacing the commonly used laser source with X‐ray, researchers obtain another powerful tool in the magnetism characterization, namely XMCD. Due to the ultrahigh photon energy of X‐ray compared to laser, XMCD can probe inner‐shell electronic transitions and electronic states that are related to magnetism, and it can also characterize the in‐plane magnetization of vdW magnets, which is the dead zone of present MOKE and MCD measurements.^[^ ^105^ ^]^ Furthermore, one can obtain element‐resolvable measurement results and analyze the spin and orbital momentum of magnetic atoms through XMCD.^[^ ^106^ ^]^ However, the demand for synchrotron radiation source may constrain its practical applications. Recently, XMCD has been applied to explore the interfacial‐hybridization‐induced and proximity‐induced magnetism in monolayer VSe_2_ via the element‐specific measurements (Figure 4f,g).^[^ ^105^, ^106^ ^]^

#### 3.1.2 PL Measurements

PL measurements have been used to probe the out‐of‐plane magnetization of vdW magnets, which can be conducted in two ways. One is to utilize linearly polarized incident light and detect both left and right circularly polarized emitted light, where the normalized difference between the intensity of these two detected lights (i.e., the circular polarization of collected lights) depends on the sample magnetization.^[^ ^113^ ^]^ Another is to use circularly polarized light as the source and detect either left or right circularly polarized luminescence, in which the average luminescence intensity of spin‐polarized states is served as the reference. Then the sample magnetization is characterized by the relative difference between the detected luminescence intensity and the reference (i.e., the relative polarization of collected luminescence).^[^ ^16^ ^]^ Compared to MOKE and MCD, it is difficult to interpret the physical mechanism of PL in the characterization of magnetism, because different relaxation processes and possible trapped states may simultaneously influence the measurement results.^[^ ^111^ ^]^

The magnetism of some Cr trihalides has been characterized by PL measurements. As for CrI_3_, the PL measurements are carried out using the first way.^[^ ^113^ ^]^ The circular polarization of collected lights is related to the magnetization direction of monolayer CrI_3_, and it will reverse with the reversal of magnetization direction (Figure 6c). Then, the pronounced hysteresis loop is a clear evidence for the FM coupling in monolayer CrI_3_ (Figure 6d). The magnetization behavior of bilayer CrI_3_ is also studied using field‐dependent measurements, which presents AFM features, coincident with the AFM interlayer coupling (Figure 6e). Further analysis by ligand‐field theory implies that the PL in CrI_3_ comes from the intraatomic d–d transition, and the electron–lattice coupling may also play a part. Moreover, PL measurements on CrI_3_/WSe_2_ heterostructures demonstrate that the proximity‐induced valley splitting occurs in WSe_2_ (Figure 6f), and further studies on valley dynamics reveal the potentials of PL measurements in studying the domain structure of vdW magnets.^[^ ^114^ ^]^ When it comes to CrBr_3_, the second way of PL measurements has been used.^[^ ^16^ ^]^ The relative polarization of collected luminescence shows the presence of ferromagnetism down to monolayer CrBr_3_ from analyzing the obvious hysteresis in the field‐dependent measurements (Figure 6g). Interestingly, these hysteresis loops are independent of collected circular polarization, which is assigned to the depolarization of output light due to the phonon scattering in the luminescence process. In addition, the magnetic anisotropy of CrBr_3_ is relatively small, promoting the formation of honeycomb/stripy domain structures in the thick samples, subsequently leading to the antiferromagnet‐like hysteresis loops in the field‐dependent PL measurements.

#### 3.1.3 Raman Measurements

**Figure 7:**
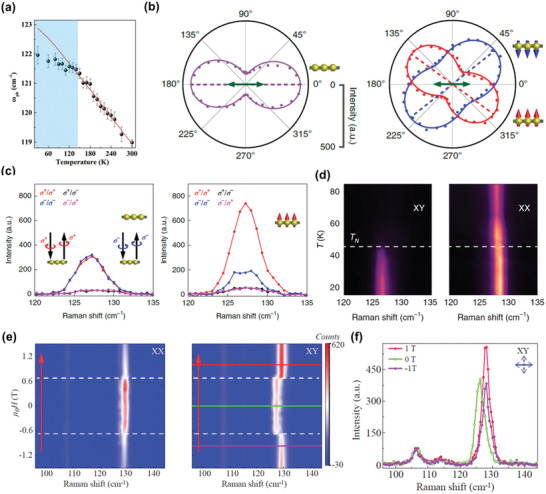
Detection of magnetism using Raman spectra. a) The temperature‐dependent phonon frequencies of *A*_1g_ mode in Fe_3_GeTe_2_ (black dots), and the corresponding fitting results using the anharmonic model (the red line). Reproduced with permission.^[^ ^117^ ^]^ Copyright 2019, Wiley‐VCH. b) The polar plots of *A*_1g_ mode in monolayer CrI_3_ at 60 K (left panel) and 15 K (right panel) with moments aligned upward (red) and downward (blue). The green arrow represents the polarization of incident light. c) The circular‐polarization resolved Raman measurements for monolayer CrI_3_ at 60 K (left panel) and 15 K (right panel) with moments aligned upward. *σ* ^+^/*σ* ^−^means that the excitation and collection are right and left circularly polarized light, respectively. d) The temperature‐dependent Raman measurements on bilayer CrI_3_ under *XY* (left panel) and *XX* configurations (right panel). Reproduced with permission.^[^ ^15^ ^]^ Copyright 2019, Springer Nature. e) The field‐dependent Raman measurements on bilayer CrI_3_ under *XX* (left panel) and *XY* configurations (right panel). f) The Raman spectra of bilayer CrI_3_ at 0 T, −1 T, and 1 T measured under *XY* configuration. Reproduced with permission.^[^ ^118^ ^]^ Copyright 2020, American Chemical Society.

The introduction of magnetic order may induce magnetic excitations^[^ ^115^ ^]^ and modulate the phonon behaviors^[^ ^62^ ^]^ of vdW magnets, resulting in phenomena like magnons,^[^ ^116^ ^]^ spin‐phonon coupling,^[^ ^72^ ^]^ and Brillouin zone folding,^[^ ^55^ ^]^ which can be detected by Raman spectrum. Compared with the methods listed above, Raman measurements are very important in the characterization of magnetism in both FM and AFM vdW magnets with in‐plane or out‐of‐plane magnetization.^[^ ^15^, ^38^, ^56^ ^]^ As follows, various magnetism‐related features can be observed in the Raman spectra of vdW magnets. The first one is the splitting of Raman characteristic peak. As for CrGeTe_3_, the peak at 76 cm^−1^ representing *E*_g_^1^ mode splits into two peaks at low temperature, due to the breaking of time‐reversal symmetry with the onset of magnetism.^[^ ^72^ ^]^ The zone‐folding effects may also lead to peak splitting as a result of magnetic ordering.^[^ ^55^ ^]^ Besides, the phonon frequency or linewidth exhibits abnormal variations near the transition temperature of vdW magnets,^[^ ^74^ ^]^ indicative of the presence of magnetism, which is related to the interaction between the exchange integral and the lattice vibration.^[^ ^72^ ^]^ From temperature‐dependent Raman measurements shown in **Figure** **7**a, it can be seen that the phonon frequency of *A*_1g_ mode in Fe_3_GeTe_2_ shows a clear deviation from the fitting line when the temperature is below *T*_C_, implying an abnormal softening of phonon with the onset of magnetism.^[^ ^117^ ^]^ In Fe_3_GeTe_2_, the lattice vibrations will modulate the superexchange interaction, leading to strong spin‐phonon coupling and later the unusual decrease of phonon frequency below *T*_C_. In addition, the presence of magnetism is also identified by the change of polarization dependence of specific Raman modes. The introduction of magnetic order modifies the Raman tensor through inducing an antisymmetric component, further altering the polarization dependence.^[^ ^118^ ^]^ Figure 7b illustrates the polarization‐dependent Raman measurements on monolayer CrI_3_. The polarization axis of *A*_1g_ mode is along the polarization direction of excitation light at the paramagnetic state, while it rotates away from the initial direction at the FM state.^[^ ^15^ ^]^ What's more, the Raman intensities excited by left circularly polarized lights may differ from those excited by right circularly polarized lights, which is also a proof for the existence of magnetism, similar to aforementioned PL measurements.^[^ ^15^, ^113^ ^]^ As shown in Figure 7c, the Raman intensities excited by left and right circularly polarized lights are almost identical for monolayer CrI_3_ above *T*_C_, while distinct intensity difference emerges below *T*_C_.

As for bilayer CrI_3_, Raman measurements have been used to characterize the transition temperature and the spin‐flip field.^[^ ^15^, ^118^ ^]^ From the symmetry analysis, the magnetism‐related phonon mode previously found in monolayer CrI_3_ will split into two modes in bilayer CrI_3_ as a result of vdW interlayer interactions, where one is classified as an odd‐parity mode (*u*) and another is an even‐parity mode (*g*).^[^ ^15^ ^]^ At the paramagnetic state, the Raman spectrum can only probe the *g* mode, because the *u* mode is infrared‐active. Thus, the Raman mode at 128.8 cm^−1^ is a *g* mode, because it is observed above the transition temperature of bilayer CrI_3_ under collinear‐polarized (*XX*) configuration (the right panel of Figure 7d). As for the *u* mode, it will become Raman‐active under cross‐polarized (*XY*) configuration at the magnetic ordered state, because the AFM interlayer coupling breaks the inversion symmetry of bilayer CrI_3_ below *T*_N_. Consequently, the transition temperature can be determined by the appearance of *u* mode in the Raman measurements under *XY* configuration. As shown in the left panel of Figure 7d, the transition temperature of around 45 K is defined by the occurrence of a Raman mode at around 126.7 cm^−1^, which is considered to be a *u* mode. Additionally, the *g* mode (128.8 cm^−1^) emerges in the field‐dependent Raman measurements under *XX* configuration, and it exhibits no distinct variation with sweeping the fields (Figure 7e).^[^ ^118^ ^]^ As for the measurements under *XY* configuration, the *u* mode (126.7 cm^−1^) exists at low magnetic fields, and then a *g* mode (128.8 cm^−1^) appears above 0.6 T (Figure 7e,f), indicating the occurrence of spin‐flip transition, which is also confirmed by the MCD measurements.^[^ ^15^, ^118^ ^]^ Compared with the AFM state, the spin‐polarized bilayer CrI_3_ restores inversion symmetry, making the *g* mode rather than the *u* mode Raman‐active under *XY* configuration above the spin‐flip field. Very recently, 2D magnons are detected in both monolayer and bilayer CrI_3_ using low‐frequency Raman spectra.^[^ ^116^ ^]^

#### 3.1.4 SHG Measurements

**Figure 8:**
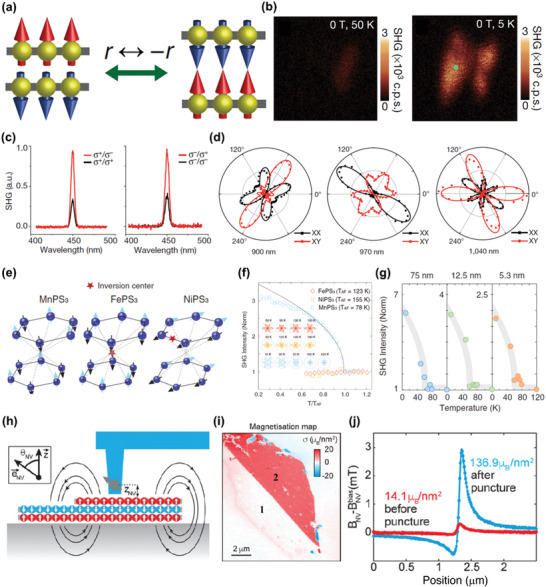
Detection of magnetism using SHG and SSSM. a) Schematic diagram of the lack of inversion center in bilayer CrI_3_ at the AFM state. Reproduced with permission.^[^ ^15^ ^]^ Copyright 2019, Springer Nature. b) The SHG intensity mapping of bilayer CrI_3_ (marked by the green dot) at 50 K (left panel) and 5 K (right panel). c) The circular‐polarization‐resolved SHG measurements on bilayer CrI_3_. d) The polar plots of SHG intensities measured on bilayer CrI_3_ using 900, 970, and 1040 nm incident laser under *XX* (black dots) and *XY* (red dots) configurations. The black line and red line are the respective fitting results using nonlinear tensors based on the monoclinic stacked structure. Reproduced with permission.^[^ ^17^ ^]^ Copyright 2019, Springer Nature. e) The illustration for the lack of inversion center in MnPS_3_ at the magnetic ordered state compared with FePS_3_ and NiPS_3_. f) The temperature‐dependent SHG intensity along the anticlockwise 60° direction relative to the horizontal axis in the polar plots of bulk MnPS_3_, FePS_3_, and NiPS_3_, and the solid line is the best fitting results using critical power law for MnPS_3_. Inset shows the polar plots of these three compounds at various temperatures. g) Temperature‐dependent SHG measurements on MnPS_3_ with different thicknesses. Reproduced with permission.^[^ ^57^ ^]^ Copyright 2020, American Physical Society. h) The schematic figure of the SSSM measurements. The gray arrow denotes the single spin in the diamond nitrogen‐vacancy, *Z*_NV_ denotes the distance between the tip and the surface, and *θ*_NV_denotes the angle of the direction of the single spin and the moments in the sample. i) The measured magnetization map of a CrI_3_ sample under the transition temperature using SSSM, where 1 and 2 denote bilayer and tri‐layer parts in this CrI_3_ sample, respectively. j) The line‐cut results of sample magnetic field across the edge of a nine‐layer CrI_3_ sample before (red line) and after puncture (blue line). Reproduced with permission.^[^ ^19^ ^]^ Copyright 2019, American Association for the Advancement of Science.

SHG is a sensitive probe for the inversion‐symmetry breaking, which has been applied to characterize vdW materials lacking inversion center.^[^ ^119^ ^]^ Specially, in some vdW magnets, the magnetic order will break the spatial inversion symmetry, making SHG measurements feasible to characterize their magnetism. Take CrI_3_ with an even number of layers as an example, its AFM interlayer coupling leads to the absence of inversion center at the magnetic ordered state (**Figure** **8**a).^[^ ^120^ ^]^ As shown in Figure 8b, the SHG intensity of bilayer CrI_3_ is negligible at the paramagnetic state, while a strong SHG signal is detected with the introduction of magnetic order.^[^ ^17^ ^]^ Besides, the polarization‐resolved SHG measurements are used to confirm the stacking order of 2D CrI_3_ at low temperature, which is possible to be rhombohedral or monoclinic according to the theoretical calculations.^[^ ^27^ ^]^ The latter is verified by the considerable SHG intensities when the excitation and collection laser have identical circular polarization (Figure 8c), because it should be nearly zero for the rhombohedral stacked structure.^[^ ^17^ ^]^ In addition, the linear‐polarization‐dependent SHG measurements are carried out on bilayer CrI_3_ using excitation lights with different wavelengths under both *XX* and *XY* configurations (Figure 8d). The results are perfectly fitted using the nonlinear tensor based on the monoclinic stacked structure, further confirming the stacking order in 2D CrI_3_. Beyond that, in vdW antiferromagnets with a Neel‐type magnetic structure, e.g., MnPS_3_ (Figure 8e), the magnetic ordering also breaks the inversion symmetry. The SHG intensity exhibits abnormal increase with the onset of magnetism in the temperature‐dependent measurements on bulk MnPS_3_, and similar phenomenon is also observed in a 5.3 nm thick MnPS_3_ flake (Figure 8f,g).^[^ ^57^ ^]^ These results affirm that MnPS_3_ lacks inversion center at the AFM state. Furthermore, from the phenomenological fitting of temperature‐dependent SHG measurement results, the critical exponent and transition temperature of MnPS_3_ are obtained. The former matches well with the Heisenberg model, and the latter is close to the value determined by Raman measurements.^[^ ^57^, ^58^ ^]^ All of these illustrate the potential of SHG measurements in the study of symmetry‐sensitive phenomena in vdW magnets.

#### 3.1.5 SSSM Measurements

The tip of SSSM is equipped with a single spin at the center of diamond nitrogen‐vacancy which serves as a nanoscale quantum probe, and it scans over the sample surface in close vicinity to read out the magnetic information (Figure 8h).^[^ ^121^ ^]^ Compared with the existing magnetic characterization methods, SSSM can provide nanoscale spatial resolution and high sensitivity, and it can also be used to quantitatively analyze the magnetism of sample. The quantitative determination of sample magnetic field is realized by the spin‐selective luminescence process of the nitrogen‐vacancy defect in diamond, which is based on the field‐dependent Zeeman splitting of spin sublevel, and the sample magnetization is further obtained through a reverse‐propagation process. These advantages endow SSSM with great significance in the characterization of vdW magnets. Recently, the magnetism of CrI_3_ is quantitatively analyzed by SSSM.^[^ ^19^ ^]^ The CrI_3_ with an odd number of layers exhibits almost identical average magnetization, close to the value of monolayer CrI_3_, confirming the interlayer AFM coupling, and it is further verified by the nearly zero magnetization in bilayer samples (Figure 8i). Interestingly, a transition from AFM to FM interlayer coupling occurs in a nine‐layer CrI_3_ after an unintentionally puncture, which is revealed by the change of average magnetization from around 14.1 to 136.9 μ_B_ nm^−2^ after the puncture (Figure 8j). This result implies that the magnetism of vdW magnets can be modulated through strain. Finally, obvious magnetic domain structures are observed in nine‐layer CrI_3_, which have quantized magnetization, namely the integer multiples of monolayer magnetization. The formation of domain structures is attributed to the spatial variation of stacking order, subsequently resulting in various numbers of FM/AFM coupled layers at different parts of the sample, which might occur in the sample preparation process.

### 3.2 Electrical Methods

#### 3.2.1 TMR‐Based Measurements

The TMR effects originate from the spin‐dependent tunneling of electrons when tunneling from one FM layer to another FM layer.^[^ ^122^ ^]^ When the magnetic moments in these two FM layers are parallel or antiparallel aligned, the tunneling resistance will have a minimum (*R*_p_) or maximum value (*R*_ap_), respectively, which provides a resistive feature for the presence of magnetism,^[^ ^122^, ^123^ ^]^ and the TMR effects can be quantified as *(R*_ap_ − *R*_p_)/*R*_p_.

**Figure 9:**
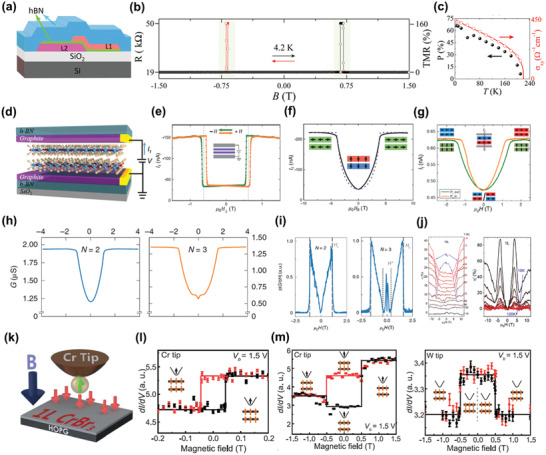
Electrical detection based on TMR. a) The schematic of an MTJ device constructed using Fe_3_GeTe_2_, where L1 and L2 represent Fe_3_GeTe_2_ flakes with different thickness. b) The measured tunneling resistance of device shown in (a) as a function of magnetic field at 4.2 K. c) The spin polarization for carriers in Fe_3_GeTe_2_ versus temperature (black) derived from the TMR measurement results, and the temperature‐dependent anomalous Hall conductivity (red dots) for Fe_3_GeTe_2_ derived from the AHE‐based measurements. The red line is the fitting results using power law. Reproduced with permission.^[^ ^124^ ^]^ Copyright 2018, American Chemical Society. d) The schematic of an MTJ device based on bilayer CrCl_3_. Reproduced with permission.^[^ ^125^ ^]^ Copyright 2019, American Chemical Society. e) The tunneling current of bilayer CrI_3_ measured under sweeping the out‐of‐plane fields. f) The tunneling current of bilayer CrI_3_ as a function of in‐plane fields. Reproduced with permission.^[^ ^22^ ^]^ Copyright 2018, American Association for the Advancement of Science. g) The tunneling current of bilayer CrCl_3_ versus out‐of‐plane and in‐plane fields. Reproduced with permission.^[^ ^125^ ^]^ Copyright 2019, American Chemical Society. h) The tunneling conductance of bilayer (left panel) and tri‐layer (right panel) CrCl_3_ measured under in‐plane magnetic fields. i) The field‐dependent d*G*/d*H* for bilayer (left panel) and tri‐layer (right panel) CrCl_3_. Reproduced with permission.^[^ ^126^ ^]^ Copyright 2019, Springer Nature. j) The TMR measured on 13‐layer (left panel) and monolayer (right panel) MnPS_3_ with out‐of‐plane magnetic fields at different temperatures. Reproduced with permission.^[^ ^127^ ^]^ Copyright 2020, American Chemical Society. k) The schematic of unique design for the SPSTM measurements. l) The measured differential tunneling conductance of H‐type stacked bilayer CrBr_3_ versus out‐of‐plane magnetic field using a Cr tip. The black data denotes the upward sweeping direction of magnetic fields, while red data represents downward. m) The field‐dependent differential tunneling conductance of R‐type stacked bilayer CrBr_3_ measured using a Cr tip (left panel) or a W tip (right panel). Reproduced with permission.^[^ ^18^ ^]^ Copyright 2019, American Association for the Advancement of Science.

When it comes to vdW ferromagnets, the TMR‐based measurements can be first conducted by using two vdW FM metals with different coercivity as the electrodes and an ultrathin *h*‐BN as the tunneling barrier, namely, constituting a magnetic tunneling junction (MTJ). The magnetic moments in these two vdW FM metals are parallel aligned at beginning, and then the TMR effects can be observed in a narrow field range with increasing the magnetic fields, where the magnetic moments in these two FM layers are antiparallel aligned due to the difference of coercivity.^[^ ^124^ ^]^ With the further increase of magnetic fields, the magnetic moments are parallel aligned again. Similar measurements have been carried out by using Fe_3_GeTe_2_ with different thicknesses as the electrodes (**Figure** **9**a). As shown in Figure 9b, the tunneling resistance has a sharp increase in a narrow field range at around ±0.7 T, meaning the change of relative alignment of magnetic moments in these two Fe_3_GeTe_2_ layers. From Figure 9b, it is known that the TMR is around 160% at 4.2 K, and the corresponding spin polarization of carriers (*P*_s_) is calculated to be 0.66. Besides, the *P*_s_ at different temperatures are determined by the TMR‐based measurements, which present essentially identical temperature dependence as the anomalous Hall conductivity obtained from the AHE measurements (Figure 9c). Such phenomenon is assigned to the dominating intrinsic contribution in AHE and the nearly temperature‐independent longitudinal resistivity in Fe_3_GeTe_2_.

TMR effects are also expected from the vdW magnets with AFM interlayer coupling and FM intralayer coupling, such as CrI_3_ and CrCl_3_.^[^ ^22^, ^125^ ^]^ These materials usually serve as the tunneling barriers, because their interlayer magnetization alignment can be effectively tuned via a small magnetic field. Afterward, graphene is also needed to act as the electrodes, due to its high conductivity (Figure 9d). As shown in Figure 9e, the tunneling current in bilayer CrI_3_ is measured under an out‐of‐plane magnetic field.^[^ ^22^ ^]^ This current is almost constant at low fields, but it shows an abrupt change to a larger value when the magnetic field is increased to a critical value. Then, the tunneling current remains unchanged at higher magnetic fields. These features demonstrate that the alignment of interlayer magnetization changes from antiparallel to parallel with increasing the magnetic fields, namely the spin‐flip transition, which is also confirmed by the MCD measurement results. The tunneling current in bilayer CrI_3_ is also detected under an in‐plane magnetic field (Figure 9f). By contrast, it increases smoothly with increasing the magnetic fields and saturates at a critical field, consistent with the out‐of‐plane magnetic anisotropy of CrI_3_. Particularly, a record high TMR of 19 000% is observed in a tetralayer CrI_3_ at low temperature. Moreover, as illustrated in Figure 9g, the TMR measurements are conducted by using bilayer CrCl_3_ as the tunneling barrier under both in‐plane and out‐of‐plane magnetic fields as well,^[^ ^125^ ^]^ which display similar field‐dependent behaviors as the TMR measurements on bilayer CrI_3_ under an in‐plane magnetic field. The saturation field is smaller when using the in‐plane magnetic field, confirming the in‐plane magnetic anisotropy of CrCl_3_. However, there is a small difference between the saturation fields when the in‐plane and out‐of‐plane magnetic fields are respectively employed, which is coincident with the relatively small magnetic anisotropy of CrCl_3_. In addition, CrCl_3_ with an odd number of layers has an extra spin‐flop transition at a low in‐plane field, which may also result from the small anisotropy.^[^ ^126^ ^]^ The field‐dependent tunneling conductance of CrCl_3_ is measured under the in‐plane magnetic field (Figure 9h), and the derivative of conductance to magnetic field (d*G/*d*H*) is also obtained, to give a better understanding of the magnetic phase transition behaviors. Because d*G/*d*H* is sensitive to the variations of relative alignment of moments in the individual layers, it will display obvious changes near the phase transition points. A sharp increase emerges near the spin‐flip field for both bilayer and tri‐layer CrCl_3_, while an extra minimum at low fields is detected for tri‐layer CrCl_3_ (Figure 9i), corresponding to the spin‐flop transition. The CrCl_3_ with an odd number of layers has an uncompensated layer which will acquire Zeeman energy from the external magnetic field. This Zeeman energy dominates at low magnetic fields, due to the small magnetic anisotropy of CrCl_3_, and it will force the magnetic moments in the whole sample to be aligned with the external magnetic field, resulting in the spin‐flop transition. Furthermore, the magnetization behavior of few‐layer CrCl_3_ determined by TMR measurements can be quantitatively analyzed using an AFM linear‐chain model.

Although not designed in the conventional manner, the magnetism of vdW antiferromagnets with AFM intralayer coupling is also studied via TMR measurements, e.g., MnPS_3_.^[^ ^127^ ^]^ In a 13‐layer MnPS_3_, the TMR is nearly field‐independent when the applied magnetic field is smaller than 5 T. Above this critical field, the TMR increases with increasing the magnetic fields, and further a steep change occurs at around 7.5 T (Figure 9j). The changes of TMR under these two magnetic fields are related to the spin‐flop transition in MnPS_3_. In bulk MnPS_3_, only one spin‐flop field exists, while two spin‐flop fields are observed in few‐layer MnPS_3_, and this splitting behavior is due to the magnetic anisotropy caused by different sources. Such field‐dependent TMR persists to the *T*_N_ of MnPS_3_, which is a clear evidence for the presence of magnetism. Strikingly, monolayer MnPS_3_ also presents obvious field‐dependent tunneling resistance, indicating the existence of magnetism down to monolayer (Figure 9j). Further work is required to understand this phenomenon, because MnPS_3_ is predicted to be a Heisenberg‐type magnet, the long‐range magnetic order of which cannot be retained in the monolayer.^[^ ^54^ ^]^

In addition to integrating into vdW heterostructures for the TMR measurements, the magnetism of vdW materials can also be directly probed by SPSTM based on the identical physical mechanism.^[^ ^128^ ^]^ For the characterization of CrBr_3_, the W tip is deposited with a Cr thin film, and then its magnetization is robust and remains stable during the measurements (Figure 9k).^[^ ^18^ ^]^ As a result, the external magnetic field only changes the magnetization direction of CrBr_3_, which will alter the relative alignment between the magnetic moments of the tip and the sample. Furthermore, the differential tunneling conductance (d*I*/d*V*) varies as well. The stacking‐dependent magnetism in CrBr_3_ has been revealed by the SPSTM measurements. The monolayer and H‐type (180° rotation between the orientation of adjacent layers) stacked bilayer CrBr_3_ exhibit normal hysteresis loops with two plateaus (Figure 9l) which correspond to two magnetization states (spin up or spin down), indicating the FM coupling between layers. The R‐type (same orientation between neighbor layers) stacked bilayer CrBr_3_ displays irregular hysteresis loops with four plateaus when using a Cr tip (Figure 9m), indicative of obvious AFM interlayer coupling. When the field is larger than 0.5 T or smaller than −0.5 T, the magnetic moments in bilayer CrBr_3_ are fully aligned with the external magnetic fields, yielding two different plateaus in the field‐dependent d*I*/d*V* measurements. In the field range of −0.5 to 0.5 T, two individual layers are AFM coupled, and another two plateaus emerge depending on how the magnetic moments in the Cr tip are aligned with the magnetic moments in the top layer of bilayer CrBr_3_. Hereafter, a bare W tip is employed to further verify the AFM interlayer coupling in bilayer CrBr_3_ holding R‐type stacking order. Similar to the TMR measurements in bilayer CrI_3_, the d*I*/d*V* curve also has plateaus with two values (Figure 9m), corresponding to the AFM and spin‐polarized states, respectively. For comparison, the d*I*/d*V* curve shows no field dependence when the bare W tip is used to characterize the bilayer CrBr_3_ possessing H‐type stacking order, which is consistent with the FM interlayer coupling.

#### 3.2.2 Hall Effect‐Based Measurements

Both NHE and AHE exist in the FM materials. The former is sensitive to the external magnetic fields, while the latter depends on the magnetization of sample.^[^ ^129^ ^]^ In vdW magnets, the Hall resistance *R_xy_* can be written as

(1) $$\begin{array}{c} R_{\mathrm{xy}}=R_{N}B_{z}+R_{A}M_{z} \end{array}$$

**Figure 10:**
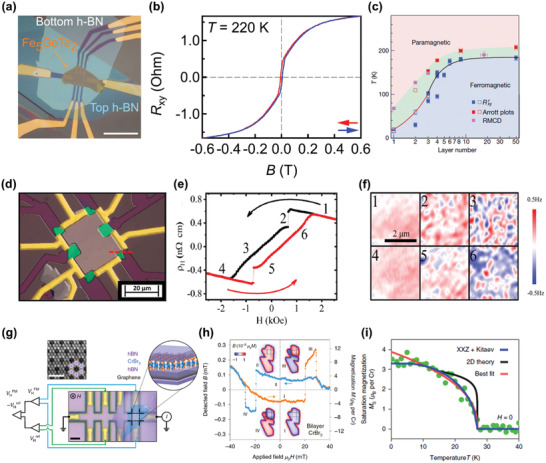
Electrical detection based on Hall effect. a) Optical micrograph of a Hall bar device made of Fe_5_GeTe_2_ to measure AHE; scale bar: 20 µm. b) The measured *R_xy_* of device in (a) versus sweeping the magnetic fields at 220 K. Reproduced with permission.^[^ ^83^ ^]^ Copyright 2019, American Chemical Society. c) The layer‐number‐temperature phase diagram of Fe_3_GeTe_2_ defined by the MCD and AHE‐based measurements. Reproduced with permission.^[^ ^21^ ^]^ Copyright 2018, Springer Nature. d) The false color optical micrograph of a Hall bar device made of CrGeTe_3_ capped by a Pt layer. The CrGeTe_3_ and Pt are denoted in green and gray, respectively. e) The anomalous Hall resistivity measured on device in (d) as a function of magnetic field. f) The characterization of Pt/CrGeTe_3_ heterostructure using MFM under different magnetic fields, corresponding to the points marked in (e). Reproduced with permission.^[^ ^131^ ^]^ Copyright 2019, American Chemical Society. g) Optical micrograph of the Hall micro‐magnetometry (scale bar: 2 µm), the measurement circuits and the schematic of the cross section of the device (top right inset). Top left inset: the atomic‐resolution structure analysis of CrBr_3_ using transmission electron microscope with the blue and yellow circles representing Cr atoms and Br atoms, respectively, and the scale bar is 1 nm. h) The measured hysteresis loop of bilayer CrBr_3_ using the Hall micro‐magnetometry. The inset shows the micromagnetic simulated domain structures at the marked points in the hysteresis loop. i) The green dots are the temperature dependence of saturation magnetization in monolayer CrBr_3_ and the solid lines are the fitting results based on different models (blue: XXZ model with Kitaev interactions, black: 2D Ising model, red: the best fitting results using the power law). Reproduced with permission.^[^ ^134^ ^]^ Copyright 2019, Springer Nature.

In Equation (1), the first term denotes the normal Hall resistance, and the second term is the anomalous Hall resistance. *B_z_* and *M_z_* are the out‐of‐plane components of applied magnetic field and the magnetization of sample, respectively. *R*_*N*_ is a coefficient that is related to the strengths of NHE, while *R*_*A*_ is a constant used to describe the strengths of AHE. In itinerant vdW ferromagnets, NHE is much weaker than AHE due to the metallic conductivity,^[^ ^130^ ^]^ so the magnetism can be characterized through the AHE‐based measurements. From Equation (1), because NHE can be ignored compared to AHE in itinerant vdW magnets, *R_xy_* is proportional to *M_z_*, thus the magnetism of these materials can be directly obtained through measuring *R_xy_* (**Figure** **10**a). As illustrated in Figure 10b, the ferromagnetism in Fe_5_GeTe_2_ is revealed by the hysteresis loop in the field‐dependent *R_xy_* measurements at 220 K.^[^ ^83^ ^]^ In addition, the ionic‐gating modulated magnetism,^[^ ^21^ ^]^ spin‐reorientation phenomena,^[^ ^81^ ^]^ and near room‐temperature ferromagnetism^[^ ^81^, ^83^ ^]^ are also observed in Fe*_y_*GeTe_2_ family through the AHE‐based measurements. What's more, compared with the optical methods that have a small spot size and detect the local magnetism, the AHE‐based measurements probe the magnetism of whole sample and will be affected by the domain structures, causing different measurement results when using optical and AHE‐based methods.^[^ ^21^ ^]^ As shown in Figure 10c, the *T*_C_ of Fe_3_GeTe_2_ obtained from the AHE‐based measurements are always lower than those from the MCD measurements, implying that the domain structures appear at the temperatures higher than the *T*_C_ determined by the AHE‐based measurements.

Through the construction of heterostructures with heavy metals or topological insulators, the AHE measurements can also be used to indirectly identify the magnetism of vdW FM insulators based on the proximity‐induced AHE (Figure 10d). As for CrGeTe_3_, clear AHE is detected from the adjacent paramagnetic Pt film, and this feature persists to the *T*_C_ of CrGeTe_3_, validating the presence of ferromagnetism.^[^ ^131^ ^]^ Besides, magnetic domain structures emerge in the CrGeTe_3_, giving rise to the slanted hysteresis loop in the field‐dependent AHE measurements and the sharp variation points in the loop (Figure 10e). As shown in Figure 10f, the magnetism of Pt/CrGeTe_3_ heterostructure is characterized using MFM at different magnetic fields (corresponding to the points marked in Figure 10e), from which clear magnetic domain structures are observed. Near the nucleation fields of domains, the hysteresis loop shows abrupt variation points (e.g., point 2). Moreover, the proximity‐induced AHE also occurs in the (Bi, Sb)_2_Te_3_/CrGeTe_3_ heterostructure, where (Bi, Sb)_2_Te_3_ is a typical topological insulator, and it disappears at the temperature higher than the *T*_C_ of CrGeTe_3_, implying that the magnetism of vdW FM insulators can be investigated using this method.^[^ ^132^, ^133^ ^]^

Apart from AHE‐based measurements, the Hall micro‐magnetometry based on NHE can also be used to characterize the ferromagnetism of vdW magnets.^[^ ^134^ ^]^ Such measurements are conducted by using a microsize Hall bar in the vicinity of magnetic materials as a probe (Figure 10g). The magnetic field of sample *B*_s_ can be obtained by analyzing the Hall response, from which one can acquire the magnetization of sample, because it is proportional to the *B*_s_. The Hall bar in this device is usually made of materials with ballistic transport properties, because these materials possess large Hall coefficient and high mobility, which guarantee high sensitivity and further quantitative analysis of the magnetization of sample.^[^ ^135^ ^]^ As for vdW materials, the ballistic transport properties have been observed in the *h*‐BN encapsulated graphene after applying a proper gate voltage,^[^ ^136^ ^]^ making it suitable for this device. Recently, the magnetism of CrBr_3_ is studied using this method.^[^ ^134^ ^]^ The ferromagnetism down to monolayer is confirmed by the pronounced hysteresis loop. Interestingly, abnormal variation points are observed in some hysteresis loops, which is attributed to the formation of fine magnetic domain structures. As shown in Figure 10h, the detected *B*_s_ from a bilayer CrBr_3_ abruptly increases to a large value at an applied field of around 20 mT when the field is swept from −40 to 40 mT, and this value is even larger than the *B*_s_ at the spin‐polarized state. Similar phenomenon is also observed at around −20 mT when the field is swept from 40 to −40 mT. From the micromagnetic simulations, it is known that such phenomena are related to the emergence of magnetic domain structures. It can be seen that the whole sample has a single domain at low fields (the insets marked I and II in Figure 10h), and a domain wall appears near the central area of Hall bar at around the critical field (the insets marked III and IV in Figure 10h). The occurrence of magnetic domains will induce a significant increase of detected *B*_s_ and the abnormal change of hysteresis loop, because the demagnetization effects decrease with the appearance of a two‐domain structure. Besides, the saturation magnetization of monolayer CrBr_3_ is obtained at different temperatures, and then its temperature dependence is fitted using various methods (Figure 10i). The temperature dependence is first fitted using the power law (red line in Figure 10i), yielding a critical exponent of around 0.4 ± 0.1, which means that the magnetization behavior in CrBr_3_ cannot be described using the 2D Ising model with a critical exponent of 0.125 (black line in Figure 10i). Furthermore, this temperature dependence is perfectly described in the whole temperature range using the anisotropic XXZ model with Kitaev interactions (blue line in Figure 10i), which is similar to CrI_3_.^[^ ^137^ ^]^

## 4 Modulation of Magnetism in vdW Magnets

Various magnetic properties of vdW magnets, such as transition temperature, magnetic anisotropy, magnetization, and coercive field, etc., have been effectively modulated through electrical, mechanical, and chemical methods. Herein, we will give a summary of existing modulation methods and their physical mechanisms.

### 4.1 Electrical Modulation

**Figure 11:**
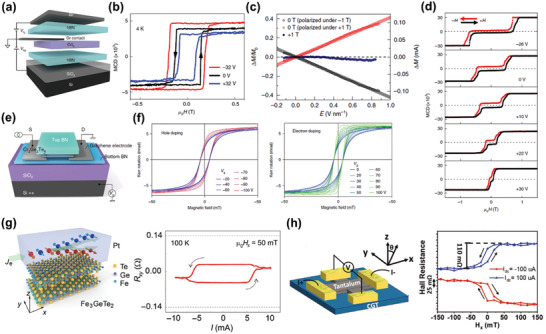
Electrical modulation of magnetism. a) The schematic of a dual‐gated device based on bilayer CrI_3_. Reproduced with permission.^[^ ^23^ ^]^ Copyright 2018, Springer Nature. b) The MCD signal of monolayer CrI_3_ versus magnetic field under electron (blue), zero (black) and hole doping (red). Reproduced with permission.^[^ ^139^ ^]^ Copyright 2018, Springer Nature. c) The variation of magnetization of bilayer CrI_3_ as a function of electrical field at zero magnetic field (red/black open circles denote that bilayer CrI_3_ is initialized by cooling below the transition temperature under a magnetic field of 1 T/−1 T, respectively) and an external magnetic field of 1 T (filled circles). Reproduced with permission.^[^ ^120^ ^]^ Copyright 2018, Springer Nature. d) The field‐dependent MCD signal of bilayer CrI_3_ under different doping levels and types (the negative gate voltage will induce hole doping, while the positive gate voltage will induce electron doping). Reproduced with permission.^[^ ^139^ ^]^ Copyright 2018, Springer Nature. e) The schematic of a field‐effect transistor made of few‐layer CrGeTe_3_ by vdW assembly. f) The field‐dependent magnetization of CrGeTe_3_ under different hole (left panel) and electron (right panel) doping levels measured by MOKE. Reproduced with permission.^[^ ^112^ ^]^ Copyright 2018, Springer Nature. g) The schematic of current control of magnetism in Fe_3_GeTe_2_ by SOT in the adjacent Pt layer (left panel) and the current switching of magnetization in Fe_3_GeTe_2_ under an in‐plane magnetic field of 50 mT characterized by AHE‐based measurements (right panel). The current direction is parallel to the magnetic‐field direction, which is along the *x*‐axis marked in the left panel. Reproduced with permission.^[^ ^146^ ^]^ Copyright 2019, American Association for the Advancement of Science. h) Left panel: the schematic of a Hall bar device made of CrGeTe_3_ capped by a Ta layer to realize the current control of magnetism. Right panel: the anomalous Hall resistance of CrGeTe_3_/Ta heterostructure as a function of in‐plane magnetic field under a current of 100 µA (blue) and −100 µA (red). The current direction is parallel to the magnetic‐field direction, which is along the *x*‐axis marked in the left panel. Reproduced with permission.^[^ ^147^ ^]^ Copyright 2020, Wiley‐VCH.

Electrical control of magnetism is of great technical significance and has been achieved in some vdW magnets by either voltage or current. The voltage control is mainly realized using electrostatic gating, causing net carrier doping and a non‐zero displacement field in the sample, which will further change the position of Fermi level and break the inversion symmetry, respectively.^[^ ^138^ ^]^ The magnetic properties of monolayer CrI_3_ are effectively modulated by carrier doping, where hole (electron) doping displays beneficial (harmful) impacts on the magnetic properties (**Figure** **11**a,b).^[^ ^139^ ^]^ For bilayer CrI_3_, the breaking of both time‐reversal and spatial‐reversal symmetry leads to the presence of linear magnetoelectric effect,^[^ ^23^, ^120^, ^140^ ^]^ which means that the magnetization can be induced by electric field and vice versa. As illustrated in Figure 11c, the magnetization of bilayer CrI_3_ shows no dependence on electric field at the spin‐polarized state, while it varies linearly with electric field at the AFM state.^[^ ^120^ ^]^ The two opposite magnetization signal is related to the two AFM configuration ↑↓ and ↓↑. Besides, the carrier doping exhibits distinct influence on the spin‐flip field of bilayer CrI_3_ (Figure 11d), which increases with hole doping and decreases with electron doping,^[^ ^23^, ^139^ ^]^ and this is interpreted by the formation of magnetic polarons with carrier doping.^[^ ^141^ ^]^ Recent studies indicate that the spin wave in bilayer CrI_3_ and the critical spin fluctuations in CrBr_3_ are effectively tuned and switched by carrier doping,^[^ ^32^, ^142^ ^]^ respectively, suggesting that electrostatic gating is a powerful tool in modulating various magnetic properties. Apart from Cr trihalides, electrostatic gating is also employed to modulate the magnetic properties of CrGeTe_3_ (Figure 11e).^[^ ^112^ ^]^ As illustrated in Figure 11f, both saturation and remnant magnetization of CrGeTe_3_ increase with the increase of gate voltages, resulting from electron or hole doping with a concentration of 10^12^ cm^−2^. Additionally, the magnetic anisotropy of CrGeTe_3_ is changed from out‐of‐plane to in‐plane by ionic gating, and the *T*_C_ is increased to above 200 K at the same time.^[^ ^143^ ^]^ Compared with electrostatic gating, ionic gating will induce electron doping with a concentration of 10^14^ cm^−2^. Under this doping level, the density functional theory calculations show that the magnetic anisotropy energy will decrease with increasing the carrier density, subsequently switching the easy axis of CrGeTe_3_. Moreover, the FM interaction mechanism of CrGeTe_3_ changes from superexchange to double‐exchange as a result of heavy carrier doping, causing the huge increase of exchange interaction and *T*_C_, which is also coincident with the theoretical calculations. These results illustrate the importance of doping level in the modulation of magnetism, and similar ionic‐gating methods have been applied to substantially improve the *T*_C_ of Fe_3_GeTe_2_.^[^ ^21^ ^]^

The current control of magnetism is achieved through the spin–orbit torque (SOT), which is exerted by the spin current in the adjacent heavy metal layers. Fe_3_GeTe_2_ exhibits high SOT efficiency in the heterostructure with a Pt film,^[^ ^144^ ^]^ where the SOT serves as an in‐plane anisotropy.^[^ ^145^ ^]^ The magnetization direction of Fe_3_GeTe_2_ is switched with increasing the current at a small in‐plane magnetic field of 50 mT (Figure 11g).^[^ ^144^, ^146^ ^]^ Besides, SOT is also used to control the magnetization of CrGeTe_3_ in a heterostructure with a Ta film. This process is conducted by applying an in‐plane magnetic field under a fixed current of around 100 µA (Figure 11h), which is much lower than non‐vdW FM materials (roughly several mA).^[^ ^147^ ^]^ The sweeping in‐plane magnetic field switches the magnetization direction of CrGeTe_3_, and the switching direction is reversed with the reversal of current direction. Furthermore, it is predicted that the switching of magnetic order in bilayer CrI_3_ can be accomplished via proximity‐induced SOT through the integration with a monolayer 1H‐TaSe_2_, which possesses both metallic conductivity and large SOC.^[^ ^148^ ^]^

### 4.2 Mechanical Modulation

**Figure 12:**
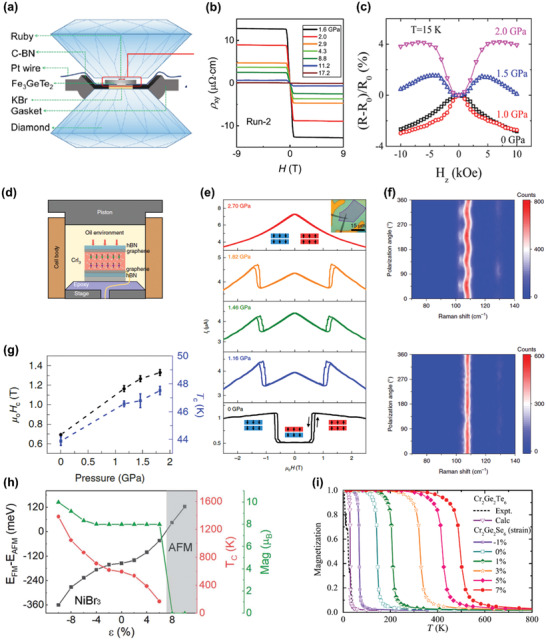
Mechanical modulation of magnetism. a) Schematic diagram of experimental setup to apply pressure on bulk Fe_3_GeTe_2_. b) The anomalous Hall resistivity of bulk Fe_3_GeTe_2_ as a function of magnetic field under different hydrostatic pressures. Reproduced with permission.^[^ ^149^ ^]^ Copyright 2019, American Physical Society. c) The field‐dependent magnetoresistance of bulk CrGeTe_3_ under different pressures. Reproduced with permission.^[^ ^75^ ^]^ Copyright 2018, American Physical Society. d) Schematic of experimental setup to apply pressure on few‐layer CrI_3_. e) The tunneling current of bilayer CrI_3_ versus sweeping magnetic fields under different pressures. Reproduced with permission.^[^ ^152^ ^]^ Copyright 2019, Springer Nature. f) The polarization dependence of Raman mode at around 107 cm^−1^ for a five‐layer CrI_3_ before (top panel) and after (bottom panel) applying pressure. Reproduced with permission.^[^ ^24^ ^]^ Copyright 2019, Springer Nature. g) The spin‐flip field and *T*_C_ of bilayer CrI_3_ measured under different pressures. Reproduced with permission.^[^ ^152^ ^]^ Copyright 2019, Springer Nature. h) The calculated strain‐dependent energy difference between the AFM and FM phases, magnetization, and *T*_C_ of monolayer NiBr_3_. Reproduced with permission.^[^ ^156^ ^]^ Copyright 2019, Royal Society of Chemistry. i) The calculated temperature‐dependent magnetization of monolayer Cr_2_Ge_2_Se_6_ under different strains. Reproduced with permission.^[^ ^26^ ^]^ Copyright 2019, American Physical Society.

The magnetism of vdW materials is strongly dependent on the structural parameters, such as bond length, bond angle, interlayer spacing, and stacking order. There is intimate correlation between electronic structure and crystal structure, making mechanical methods that can modify crystal structures effective in controlling the magnetic properties of vdW magnets. Hydrostatic pressure is a powerful tool in modulating the magnetism of many vdW magnets. The anomalous Hall resistivity of bulk Fe_3_GeTe_2_ decreases with increasing the pressure (**Figure** **12**a,b), indicating the weakening of magnetism.^[^ ^149^ ^]^ It may arise from the pressure‐induced shift of splitting band and the suppression of magnetic moment under pressure. The pressure will also weaken the FM superexchange interaction in bulk CrGeTe_3_, further decreasing its *T*_C_.^[^ ^150^ ^]^ From the Goodenough‐Kanamori‐Anderson rules, it is known that the 90° magnetic‐ion‐ligand‐magnetic‐ion angle favors FM superexchange interaction, while the 180° angle benefits AFM superexchange interaction. As for CrGeTe_3_, it is predicted that the Cr—Te—Cr bond angle deviates from 90° with increasing the pressure, which will result in the decrease of FM superexchange interaction and *T*_C_. Besides, the magnetic anisotropy of bulk CrGeTe_3_ is switched from out‐of‐plane to in‐plane at around 2 GPa.^[^ ^75^ ^]^ As illustrated in Figure 12c, the magnetoresistance of bulk CrGeTe_3_ is measured using an out‐of‐plane magnetic field under different pressures. It shows no saturation at 0 GPa, while a clear saturation behavior is observed at 2 GPa. From this, it can be seen that the magnetization direction of sample is collinear with the direction of applied field at 0 GPa, while it differs from the initial direction when the pressure is increased to 2 GPa. Furthermore, a magnetic field of around 6 kOe is needed to saturate the magnetization of sample at 2 GPa, which is much larger than the demagnetizing field of bulk CrGeTe_3_ (≈1.8 kOe). This result indicates that the magnetization direction of bulk CrGeTe_3_ is in‐plane at 2 GPa. According to the first‐principles calculations, such spin‐reorientation behavior results from the reduction of vertical Cr—Te bond length with increasing the pressure, which will modify the magnetic anisotropy energy and arouse the switching of easy axis afterward.

More attention is also paid to Cr trihalides, whose magnetism strongly depends on the stacking order at 2D limit,^[^ ^18^, ^27^ ^]^ making pressure a powerful tool to control their magnetism. In 2D CrI_3_, it is predicted that monoclinic stacking order causes AFM interlayer coupling, while rhombohedral stacking order leads to FM interlayer coupling, originating from the direct‐exchange interaction mechanism of interlayer coupling.^[^ ^27^, ^151^ ^]^ Recently, the magnetism of 2D CrI_3_ is successfully modulated via pressure (Figure 12d).^[^ ^24^, ^152^ ^]^ Figure 12e shows the field‐dependent tunneling current measurement results on bilayer CrI_3_ under different pressures. The pressure of around 2.7 GPa switches AFM interlayer coupling to FM, which is evidenced by the disappearance of spin‐flip transition in bilayer CrI_3_.^[^ ^152^ ^]^ The tunneling current decreases steadily with increasing the fields at 2.7 GPa, and it is ascribed to the magnetoresistance from the graphene electrodes. Meanwhile, the pressure also changes the stacking order of bilayer CrI_3_, which is confirmed by the Raman measurements. From Figure 12f, it can be seen that the fourfold and polarization‐independent Raman patterns are obtained before and after applying pressure, which correspond to the monoclinic and rhombohedral stacked structure, respectively.^[^ ^152^, ^153^ ^]^ In addition, both the spin‐flip field and *T*_C_ of few‐layer CrI_3_ are increased as the pressure increases (Figure 12g), which is interpreted by the enhancement of interlayer and intralayer coupling, respectively.^[^ ^152^ ^]^ The pressure will decrease the interlayer spacing, thus increasing the interlayer coupling, and it will also strengthen the FM superexchange interaction and later the intralayer coupling, because the Cr—I—Cr bond angle of CrI_3_ will approach 90° with the increase of pressure.^[^ ^152^, ^153^ ^]^ Moreover, few‐layer CrBr_3_ also exhibits stacking‐dependent magnetism,^[^ ^18^ ^]^ making pressure feasible in the modulation of its magnetism.

In addition to pressure control, the strain modulation has also been applied to modify the structure parameters, and further control the optical properties^[^ ^154^ ^]^ and structural phase transitions^[^ ^155^ ^]^ of vdW materials. On the subject of vdW magnets, numerous theoretical works are carried out to explore the strain modulation of various magnetic properties. From Figure 12h, it is observed that NiBr_3_ will experience a magnetic phase transition from FM to AFM phases under a biaxial tensile strain of around 7%.^[^ ^156^ ^]^ Besides, the *T*_C_ of Cr_2_Ge_2_Se_6_ is increased from 144 to 421 K by applying a tensile strain of 6% (Figure 12i).^[^ ^26^ ^]^ Such strain modulation arises from the modification of crystal structure, which will subsequently influence the band structure and the exchange interaction strengths as well. Very recently, the magnetism of few‐layer CrI_3_ is effectively modulated via strain using a nanoelectromechanical system.^[^ ^157^ ^]^ The strain weakens the interlayer exchange interaction and later decreases the spin‐flip field of few‐layer CrI_3_.

### 4.3 Chemical Modulation

**Figure 13:**
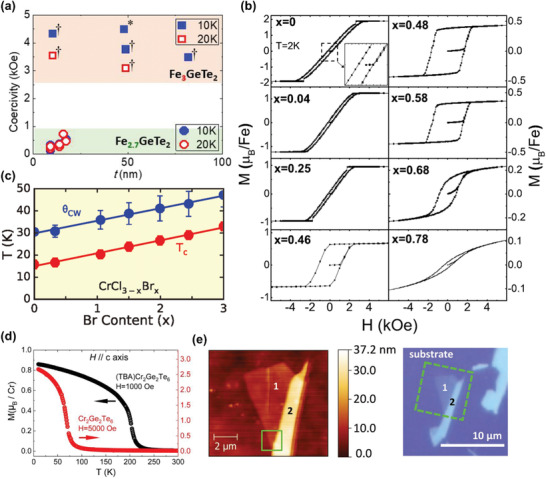
Chemical modulation of magnetism. a) The coercivity of Fe_3_GeTe_2_ (rectangular) and Fe_2.7_GeTe_2_ (circle) with different thicknesses at 10 (blue) and 20 K (red). Reproduced with permission.^[^ ^79^ ^]^ Copyright 2019, American Chemical Society. b) The magnetic moment of bulk (Fe_1−_ *_x_*Co*_x_*)_3_GeTe_2_ versus out‐of‐plane magnetic fields at different Co contents. Reproduced with permission.^[^ ^80^ ^]^ Copyright 2019, American Physical Society. c) The Curie–Weiss temperature and *T*_C_ of bulk CrCl_3−_ *_x_*Br*_x_* as a function of Br content. Reproduced with permission.^[^ ^25^ ^]^ Copyright 2018, Wiley‐VCH. d) The temperature‐dependent magnetization of CrGeTe_3_ measured under an external magnetic field of 5000 Oe (red), and the temperature‐dependent magnetization of tetra‐butyl ammonium intercalated CrGeTe_3_ measured under a magnetic field of 1000 Oe (black). Reproduced with permission.^[^ ^165^ ^]^ Copyright 2019, American Chemical Society. e) The atomic force microscopic image (left panel) and optical microscopic image (right panel) of an exfoliated CrCl_3−_ *_x_*Br*_x_* flake. Reproduced with permission.^[^ ^25^ ^]^ Copyright 2018, Wiley‐VCH.

As for traditional magnetic materials with finite phase width and isostructural compounds, methods like changing stoichiometric ratio and elemental substitution have been widely used to modulate their magnetism.^[^ ^158^, ^159^, ^160^ ^]^ Some members of vdW magnet family also possess finite phase width, making these two methods feasible in the modulation of magnetism. As above mentioned, the Fe content in Fe*_y_*GeTe_2_ can be adjusted in a finite range,^[^ ^161^ ^]^ resulting in the synchronous change of magnetic properties. Take Fe_3_GeTe_2_ as an example, the magnetism is affected by the Fe content,^[^ ^162^ ^]^ and the Fe deficiency significantly decreases the *T*_C_ and coercivity in thin flakes. As shown in **Figure** **13**a, the coercivity of Fe_2.7_GeTe_2_ is below 1 kOe, while it is above 3 kOe in Fe_3_GeTe_2_.^[^ ^79^ ^]^ The magnetic anisotropy of Fe_3_GeTe_2_ decreases with lowering the Fe content, arousing the reduction of coercivity, which is a result of the variation of electronic structure that is related to the magnetic anisotropy. Meanwhile, elemental substitution also affects the magnetism of bulk Fe*_y_*GeTe_2_. The Co substitution increases the domain wall energy, further improving the coercivity of bulk Fe_3_GeTe_2_ (Figure 13b).^[^ ^80^ ^]^ In contrast, Ni doping will suppress the ferromagnetism of Fe_3_GeTe_2_ due to the dilution effect of Ni, as indicated by the decrease of *T*_C_ and effective moment.^[^ ^163^ ^]^ Likewise, the magnetic properties of bulk Fe_5_GeTe_2_ are modulated via Ni substitution as well, and it changes the bond length and interlayer spacing, subsequently enhancing the saturation moment of Fe_5_GeTe_2_.^[^ ^84^ ^]^ Apart from Fe*_y_*GeTe_2_, elemental substitution is also useful in modulating the magnetism of other vdW magnets. The Br substitution in bulk CrCl_3_ will cause a linear increase of *T*_C_ with Br content (Figure 13c), and it will switch the magnetic anisotropy from in‐plane to out‐of‐plane as well.^[^ ^25^ ^]^ These are interpreted by the modification of superexchange interaction. Then, it is predicted that the Mn substitution in monolayer VI_3_ will lead to a spin half metal state.^[^ ^164^ ^]^ Besides, the weak interlayer vdW interaction enables chemical intercalation to tune the magnetic properties of vdW magnets. As for bulk CrGeTe_3_, the intercalation of tetra‐butyl ammonium will induce heavy electron doping and modify the FM exchange mechanism, resulting in the increase of *T*_C_ from 67 to 208 K (Figure 13d),^[^ ^165^ ^]^ similar to the aforementioned ionic‐gating modulation.^[^ ^143^ ^]^ What's more, the Na intercalation substantially increases the *T*_C_ of bulk Fe_3_GeTe_2_, yet it is proved that this modulation arises from Fe_2−_ *_x_*Ge impurities.^[^ ^166^ ^]^ Although most studies of chemical modulation are carried out on bulk samples, few‐layer chemically modified samples have also been obtained by mechanical exfoliation (Figure 13e),^[^ ^25^ ^]^ and therefore further researches to the 2D limit are expected.

## 5 Perspective of vdW Magnets in Spintronics

**Figure 14:**
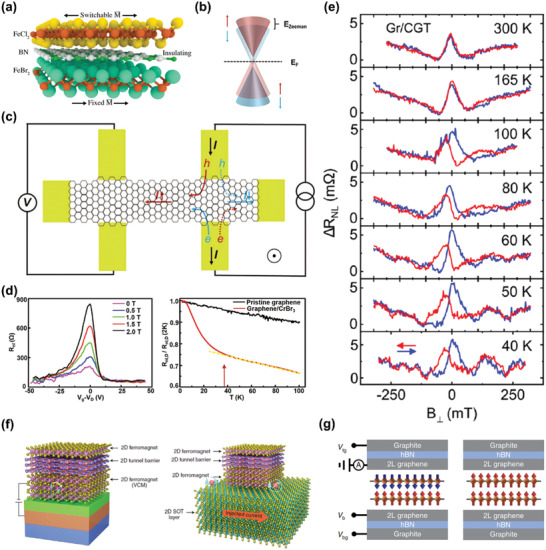
Perspective of vdW magnets in spintronics. a) Schematic diagram of the MTJ using half‐metallic vdW magnets as electrodes. Reproduced with permission.^[^ ^42^ ^]^ Copyright 2017, American Chemical Society. b) The schematic of the spin splitting of band structures in monolayer graphene. The red and blue arrows denote different spin directions of carriers. c) The schematic of the nonlocal transport measurements in graphene. The carriers with different spins are distinguished by the color (blue and red). There is no transverse charge current but exists net spin current. d) Left panel: the *R*_nl_ of monolayer graphene proximitized with CrBr_3_ as a function of gate voltage at different external magnetic fields. The Dirac point is denoted by the zero point in the abscissa axis. Right panel: the temperature‐dependent *R*_nl,D_ of pristine graphene (black) and graphene proximitized with CrBr_3_ (red). The *T*_C_ of CrBr_3_ is marked by the red arrow. Reproduced with permission.^[^ ^171^ ^]^ Copyright 2020, Wiley‐VCH. e) Field‐dependent nonlocal Hanle spin precession measurements for the graphene proximitized with CrGeTe_3_ at different temperatures. Reproduced with permission.^[^ ^174^ ^]^ Copyright 2019, IOP Publishing Ltd. f) Schematic of voltage torque‐MRAM (left panel) and SOT‐MRAM (right panel) made of magnetic vdW materials. Reproduced with permission.^[^ ^170^ ^]^ Copyright 2019, Springer Nature. g) Schematic of spin tunneling field‐effect transistors made of bilayer CrI_3_. Reproduced with permission.^[^ ^177^ ^]^ Copyright 2019, Springer Nature.

The generation, transportation, and manipulation of spin are three essential functionalities for the fabrication of spintronic devices and circuits. When it comes to vdW materials, graphene possesses excellent spin transport properties due to its small SOC and high mobility.^[^ ^167^ ^]^ As a comparison, the large SOC in TMDs (MoS_2_, MoSe_2_, WS_2_, WSe_2_) has been applied to manipulate the spin in adjacent non‐vdW FM layer through SOT.^[^ ^168^, ^169^ ^]^ The vdW magnets are essential for fabricating all‐vdW spintronic devices, where it will play an important role in the spin generation. Itinerant vdW magnets such as Fe*_y_*GeTe_2_ can act as spin sources to generate highly spin‐polarized current,^[^ ^81^ ^]^ and the polarization can be further increased to 100% by using half‐metallic vdW magnets (**Figure** **14**a).^[^ ^44^ ^]^ The injection of spin into graphene is an indispensable procedure in spintronic devices and circuits. As for itinerant and half‐metallic vdW magnets, spin injection is realized by the direct contact with graphene, and the injection efficiency can be further enhanced by the optimization of interface resistance.^[^ ^170^ ^]^ For vdW magnetic insulators, the MPE can be employed to inject spin into graphene. In the graphene/CrBr_3_ heterostructure, the spin injection is confirmed by the considerable Zeeman spin Hall effect (ZSHE) in graphene.^[^ ^171^ ^]^ Due to the MPE, the electronic band of different spin directions will split (Figure 14b), which will generate carriers with opposite spins and induce ZSHE in graphene. As shown in Figure 14c, a small longitudinal current *I* is applied in the right part of graphene at first. Under an out‐of‐plane magnetic field, the electrons and holes will have opposite flowing directions because of the Lorentz force, resulting in zero transverse charge current in graphene. However, non‐zero spin current exists as a result of MPE. Due to the long spin‐diffusion length in graphene, this spin current can be detected using the nonlocal transport measurements. Under the configuration illustrated in Figure 14c, the nonlocal voltage (*V*_nl_) can be directly measured in the left part of graphene, then one can obtain the nonlocal resistance *R*_nl_ *= V*_nl_/*I*. As shown in Figure 14d, the information about ZSHE can be acquired from the *R*_nl_ at the Dirac point (*R*_nl_ *_,_*_D_). The *R*_nl_ *_,_*_D_ exhibits significantly increases with the increase of magnetic fields, suggesting the presence of sizable ZSHE and the successful injection of spin into graphene. Through temperature‐dependent *R*_nl_ *_,_*_D_ measurements, one can further validate the spin injection. As displayed in Figure 14d, the *R*_nl,D_ varies drastically in the graphene/CrBr_3_ heterostructure below the *T*_C_ of CrBr_3_, and it exhibits little temperature‐dependence at higher temperature. In contrast, the *R*_nl,D_ of individual graphene shows no variations as the temperature increases. It is concluded that the MPE from CrBr_3_ is the main contribution for the *R*_nl,D_ below *T*_C_, that is, the spin injection is achieved via the MPE, and these phenomena are attributed to the spin‐dependent interlayer interaction with CrBr_3_.^[^ ^172^ ^]^ In addition, the single magnon‐assisted tunneling process in CrBr_3_ can also be utilized to inject spin into graphene with the conservation of momentum.^[^ ^173^ ^]^ Apart from CrBr_3_, the spin transport and precession in monolayer graphene is manipulated in the heterostructure with CrGeTe_3_ as well,^[^ ^174^ ^]^ which is characterized using nonlocal Hanle spin precession measurements. Such measurements are conducted using a nonlocal spin valve configuration under sweeping the out‐of‐plane magnetic fields. As shown in Figure 14e, conventional Hanle signals are observed in the graphene/CrGeTe_3_ heterostructure at room temperature, arising from the Larmor precession of spins. However, at low temperatures, the Hanle signal presents features like peak splitting and peak intensity difference under the magnetic field with different sweeping directions. The CrGeTe_3_ will induce an exchange field in graphene, and its hysteretic feature causes the peak splitting phenomena. Besides, the spatial distribution of this field is inhomogeneous, arousing the peak intensity difference under different sweeping fields.

Another important application of vdW magnets is the construction of MTJ. As above mentioned, the magnetism of vdW magnets can be probed using this device. It is also an important component of modern spintronic industry, based on which the memory and logic devices can be developed. The present vdW magnet‐based MTJs are composed of spin valves^[^ ^124^ ^]^ and spin‐filter MTJs.^[^ ^22^ ^]^ The former relies on itinerant vdW ferromagnets to act as the junction electrodes, and the latter derives from vdW magnets possessing AFM interlayer coupling and FM intralayer coupling. However, the switching of these MTJs mainly depends on the external magnetic fields, which is detrimental to the practical applications, and thus all‐electric switching is of technical significance. As for present spintronic industries, spin‐transfer‐torque (STT) and SOT are two promising methods for the electrical switching of MTJ using current. STT switching has not been reported in vdW magnets, and we think insufficient studies on exchange bias and relatively large current to achieve the magnetization switching may be two obstacles. But SOT switching has been realized in Fe_3_GeTe_2_ and CrGeTe_3_ under a small in‐plane magnetic field in the heterostructure with a heavy metal layer.^[^ ^146^, ^147^ ^]^ It can be further utilized to fabricate memory devices with MTJ, namely SOT magnetoresistive random access memory (SOT‐MRAM). Hereafter, an all‐vdW SOT‐MRAM can be obtained by replacing heavy metal with vdW topological insulators or TMDs which possess large SOC (Figure 14f).^[^ ^168^, ^175^ ^]^ However, further works are necessary to conceive a delicate device structure to remove the in‐plane magnetic field to realize all‐electric switching.^[^ ^176^ ^]^ Particularly, the magnetization of bilayer CrI_3_ can also be switched by the gate voltage under a small out‐of‐plane magnetic field,^[^ ^23^ ^]^ enabling the electrical switching by voltage and the fabrication of spin tunneling field‐effect transistors (Figure 14g).^[^ ^177^ ^]^ Actually, this voltage switching of magnetization, together with aforementioned magnetic anisotropy switching^[^ ^143^ ^]^ by ionic gating, offer exciting opportunities for the construction of next‐generation voltage‐torque MRAM (Figure 14f),^[^ ^170^, ^178^ ^]^ which is more energy efficient than SOT/STT‐MRAM that relies on current. Moreover, other external stimuli such as strain can also switch the magnetization of vdW magnets,^[^ ^156^ ^]^ which provides novel strategies to design the memory devices, e.g., the construction of artificial multiferroic devices with vdW ferroelectrics.^[^ ^179^ ^]^

## 6 Summary and Outlook

In summary, magnetic vdW materials are a large family, and there are still many members under exploration. The number of vdW magnets will grow rapidly in the near future. Although most attention has been paid to ferromagnets, the significance of antiferromagnets cannot be ignored, because the exchange bias effect at the interface of vdW ferromagnet and antiferromagnet is crucial in the fabrication of all‐vdW memory devices. The vdW antiferromagnets also hold great potentials in the application of nonvolatile, energy‐efficient spintronic devices.^[^ ^180^ ^]^ Besides, some vdW magnets possess novel properties such as half‐metallicity and multiferroicity, opening up an exciting plethora for the construction of multifunctional vdW architectures.^[^ ^43^, ^44^ ^]^ Moreover, the vdW magnets under investigation mainly consist of metals and insulators, while vdW magnetic semiconductors are also very important, because these materials can be utilized for the generation of highly polarized spin current and the building of devices like spin field‐effect transistors. In addition, most currently used detection tools can only give qualitative descriptions on the magnetism of vdW magnets, and more quantitative methods are expected to acquire in‐depth understanding of 2D magnetism. Specially, novel detection techniques are required in the characterization of vdW antiferromagnets, such as anisotropic magnetoresistance measurements.^[^ ^111^ ^]^ As for the modulation of magnetism in vdW magnets, more attention is needed in the effective switching of magnetization direction, magnetic anisotropy, and net magnetic moment of ferromagnets or antiferromagnets. These will exhibit distinct on/off states, which is important in the practical spintronic applications. Several methods have been applied to switch the magnetization of vdW ferromagnets,^[^ ^120^, ^143^, ^146^ ^]^ and external perturbations such as electrical‐field and strain may be effective to switch the magnetism of vdW antiferromagnets with relatively small magnetic anisotropy.^[^ ^181^ ^]^

## Conflict of Interest

The authors declare no conflict of interest.
